# Liposomes for malaria management: the evolution from 1980 to 2020

**DOI:** 10.1186/s12936-021-03858-0

**Published:** 2021-07-27

**Authors:** Patrick B. Memvanga, Christian I. Nkanga

**Affiliations:** Faculty of Pharmaceutical Sciences, Laboratory of Pharmaceutics and Phytopharmaceutical Drug Development, University of Kinshasa, B.P. 212, Kinshasa XI, Democratic Republic of the Congo

**Keywords:** Malaria, Anti-malarial-based liposomes, Liposomal malaria antigens, Targeting delivery, In vitro and in vivo anti-malarial activity

## Abstract

Malaria is one of the most prevalent parasitic diseases and the foremost cause of morbidity in the tropical regions of the world. Strategies for the efficient management of this parasitic infection include adequate treatment with anti-malarial therapeutics and vaccination. However, the emergence and spread of resistant strains of malaria parasites to the majority of presently used anti-malarial medications, on the other hand, complicates malaria treatment. Other shortcomings of anti-malarial drugs include poor aqueous solubility, low permeability, poor bioavailability, and non-specific targeting of intracellular parasites, resulting in high dose requirements and toxic side effects. To address these limitations, liposome-based nanotechnology has been extensively explored as a new solution in malaria management. Liposome technology improves anti-malarial drug encapsulation, bioavailability, target delivery, and controlled release, resulting in increased effectiveness, reduced resistance progression, and fewer adverse effects. Furthermore, liposomes are exploited as immunological adjuvants and antigen carriers to boost the preventive effectiveness of malaria vaccine candidates. The present review discusses the findings from studies conducted over the last 40 years (1980–2020) using in vitro and in vivo settings to assess the prophylactic and curative anti-malarial potential of liposomes containing anti-malarial agents or antigens. This paper and the discussion herein provide a useful resource for further complementary investigations and may pave the way for the research and development of several available and affordable anti-malarial-based liposomes and liposomal malaria vaccines by allowing a thorough evaluation of liposomes developed to date for the management of malaria.

## Background

Malaria is a life-threatening infectious disease caused by *Plasmodium falciparum*, *Plasmodium malariae*, *Plasmodium ovale*, *Plasmodium vivax* and/or *Plasmodium knowlesi*, which mostly affect people in over 87 countries located in tropical and subtropical regions of the world. Malaria has been recognized as one of the leading causes of illnesses and deaths in the world [1]. Indeed, during the last decade, malaria caused between 210 and 260 million clinical episodes and up to 400,000 deaths annually. In 2020 alone, 94% of the malaria cases were recorded in the sub-Saharan Africa. Nigeria (27%), the Democratic Republic of the Congo (12%), Uganda (5%), Mozambique (4%) and Niger (3%) accounted for about 51% of all malaria cases golobally [1]. Children of less than 5 years and pregnant women living in these regions account approximately 85% of deaths [1].

The treatment of malaria is essentially based on a series of drugs recommended by the World Health Organization (WHO) or those adopted by the government of the various endemic countries [2]. However, most of the drugs used to treat malaria are subject to therapeutic failures and resistance of *Plasmodium* species [3–6]. The factors that contribute to these shortcomings include the intrinsic drawbacks of anti-malarial drugs such as low bioavailability (poor aqueous solubility, permeability and/or biostability) and critical adverse side effects that result in poor patients’ compliance [7]. To address these bottlenecks, nanotechnology-based drug delivery systems have emerged as important therapeutic tools in the management of malaria [7–9]. Indeed, the benefits of drug delivery nanotechnology include the enhancement of efficacy, the reduction of unwanted toxic side effects, the significant improvement in patients’ compliance, and the overcoming of drug resistance development. In addition, nano-drug delivery systems may provide cell adhesion abilities and properties to conjugate specific ligands on their surface leading to passive or selective active targeting of drugs at the site of the disease [7, 8, 10].

Nanodelivery systems are composed of nanocarriers that are particulate dispersions or solid colloidal structures ranging generally from 1 to 1000 nm in diameter. These nanoparticles consist of polymeric, lipid or inorganic materials, within which the active pharmaceutical agents can be dissolved, encapsulated, absorbed and/or chemically attached [7–9]. The present review highlights the data reported within the period of 1980–2020, focusing on the findings assessing the biological performances and/or efficacies of a type of nano-platform: liposomes, a lipidic nanocarrier that holds a great potential for improving the therapeutic outcome of existing and emerging drugs against *Plasmodium* infections. This review also provides some background on the malaria life cycle, chemoprophylaxis, and chemotherapy, as well as key references for interested readers. As a result, this review may pave the way for additional research as well as the development of several readily available and affordable anti-malarial-based liposomes and liposomal malaria vaccines.

## Malaria

### The *Plasmodium spp* life cycle

**Fig. 1:**
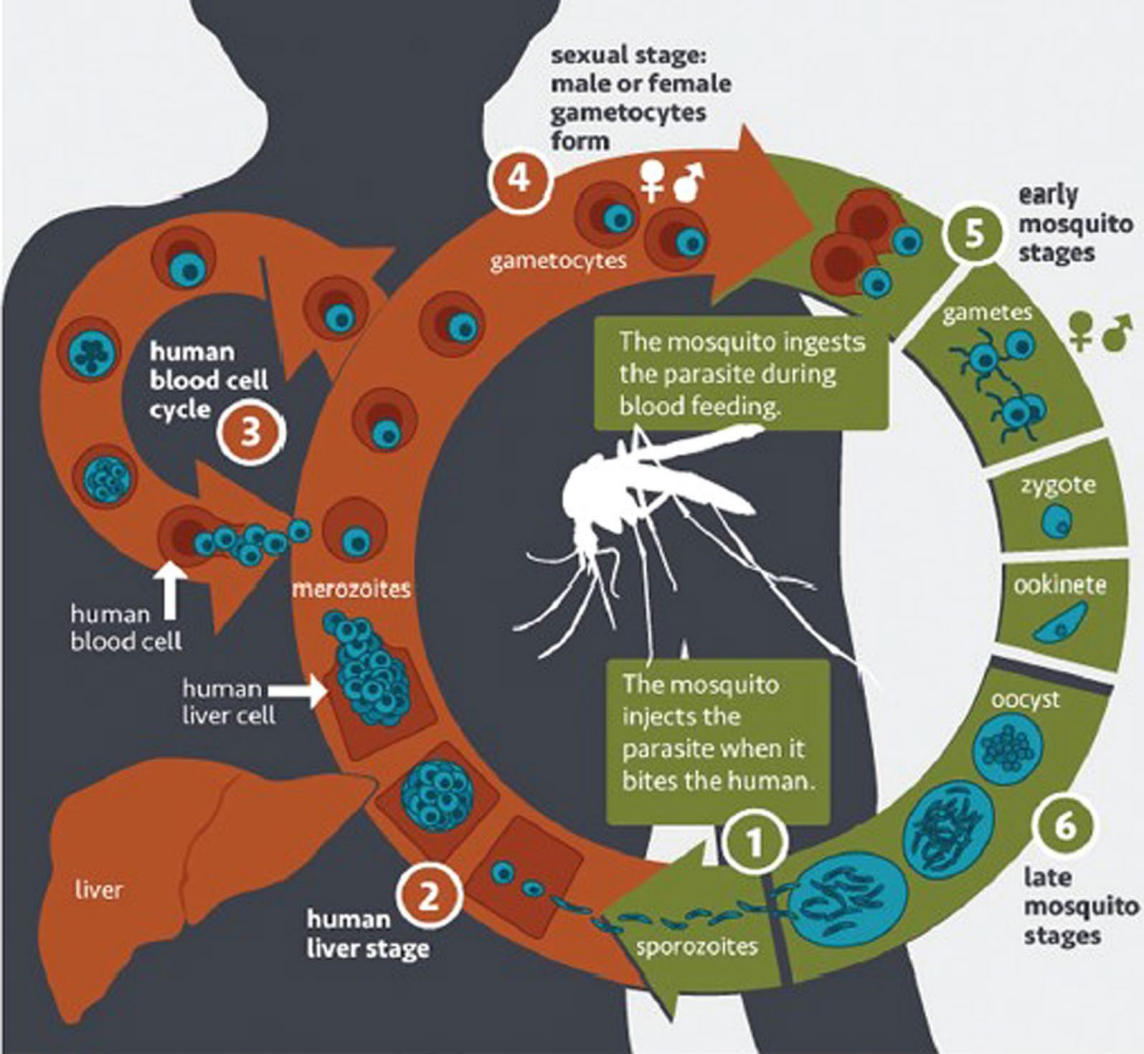
Schematic representation of the life cycle of the malaria parasite. Merozoites released from human liver and blood cells can either invade new erythrocytes (stage 3) or differentiate into gametocytes (stage 4). In their intraerythrocytic cycle, merozoites evolve into ring trophozoites, mature trophozoites and then schizonts (which consist of many daughter merozoites) [15, 16]. Image courtesy: National Institute of Allergy and Infectious Diseases (https://www.niaid.nih.gov/diseases-conditions/malaria-parasite)

As shown in Fig. 1, malaria starts with the inoculation of sporozoites from *Anopheles* mosquitoes into the human skin (dermis) [11]. While a minority (~ 20%) of these inoculated sporozoites moves randomly to the lymphatic system, 80% of them travel to the liver [12, 13]. In the hepatocytes, the sporozoites evolve a mature form known as liver schizonts. These schizonts undergo mitosis to produce exo-erythrocytic merozoites (tissue schizogony or liver stage) [11]. Following replication within hepatocytes, mature merozoites are released into the blood circulation [14].

In general, the malaria-liver stage takes 7–10 days [14]. However, in the case of *P. vivax* and *P. ovale* infections, some liver schizonts turn into hypnozoites, a dormant stage that, if untreated, can persist in the liver for months or even years [17, 18]. Afterward, hypnozoites can reactivate into schizonts causing malaria relapses by invading the bloodstream in the absence of an infectious mosquito bite [17, 18]. In 2017, the *P. vivax* parasite was responsible for approximately 7.4 million cases of malaria worldwide, 82% of which was recorded in Afghanistan, Ethiopia, India, Indonesia and Pakistan [19].

Mature merozoites that invade erythrocytes evolve into early trophozoites (ring stage) [16]. Feeding on haemoglobin and plasma nutrients, the parasites grow in mature trophozoites (trophozoite stage). These trophozoites replicate their DNA to develop into intra-erythrocytic schizonts, consisting of many daughter merozoites [15, 20].

After approximately eight division cycles, the rupture of schizonts occurs releasing the merozoites in the bloodstream. The latter enter other erythrocytes to perpetuate the blood-stage cycle, thereby increasing exponentially the parasite biomass that can exceed >20–30% in *P. falciparum* infection [21, 22]. The time for one replication cycle is 48 h for *P. falciparum*, *P. vivax* and *P. ovale*, whereas *P. malariae* and *P. knowlesi* display a 72-h and a 24-h asexual life cycle, respectively [23]. In erythrocytes, the *Plasmodium* parasites induced physical and chemical stress that result in programmed erythrocyte death, termed eryptosis (erythrocyte apoptosis) [24]. After several cycles, some of the merozoites differentiate into female or male gametocytes (sexual erythrocytic stage). When the *Anopheles* mosquitoes feed with the blood meal, gametocytes reach their guts where they develop in new sporozoites passing through the zygote, ookinete and oocyst stages (sexual mosquito stage) [15, 25].

The sporozoites as well as the liver and the sexual stages of the parasite life cycle are clinically silent. All the clinical signs and symptoms are associated with the erythrocytic phase of the infection [21, 25]. Also, unlike other forms of human malaria, falciparum malaria results in a range of outcomes from asymptomatic infection through moderate to severe malaria [26, 27]. This progression may occur within a few hours and depends on the entomological inoculation rate, the number of falciparum sporozoites inoculated and the level of immunity acquired through previous exposures [28, 29]. While the pathogenesis of uncomplicated malaria includes periodic fevers, chills, perspiration, headaches and myalgia, that of severe malaria is characterized by microvascular obstruction, hypoxia, hypoglycaemia, metabolic acidosis, cardiac insufficiency, anaemia, jaundice, renal failure, cerebral oedema and acute respiratory failure. In severe malaria, the case fatality is typically 10–20% [30–35]. The onset of severe falciparum malaria can also be due to delayed, incomplete or inappropriate treatments. Therefore, the efficacy of anti-malarial drugs as well as the speed of therapeutic response is of important considerations [27].

### Chemoprophylaxis and chemotherapy of malaria

To control malaria vectors and therefore outbreaks of malaria, insecticide-treated nets and indoor residual spraying are the most frequent preventative measurements [36–38]. Insecticide-treated (bed) nets prevent mosquitoes from biting humans and feeding on their blood, thus preventing new infections and reducing the vector population [39–41]. In addition to their ability to act as a protective barrier around people sleeping under them, mosquito nets can also kill *Anopheles* that land on them [39–41]. However, the downsides of insecticide-treated bed nets are that they would delay the onset of immunity, which, as stated above, depends on the number and rate of mosquito bites.

On the other hand, indoor residual spraying is the regular application of chemical insecticides to household’s walls and other surfaces [37]. One of the drawbacks of this measurement is the fact that the percentage of sprayed surface per dwelling should exceed 80% [37, 42]. Another weakness is that the duration of effective action of the insecticides used is on average 6 months, which requires repeated pulverization (about 2 per year) [37]. Finally, the resistance of mosquitoes to the pyrethroid insecticides used represents a common challenge for the two strategies (i.e. insecticide-treated nets and indoor residual spraying) [36, 43]. Consequently, research efforts are invested in this field to reduce malaria transmission through the mosquito in order to decrease malaria prevalence locally or globally. In this context, the nanotechnology based on green synthesis from plants having mosquito repellent activities plays an important role [44, 45].

Based on their antiplasmodial activity in the targeted life cycle stages of the *Plasmodium* species, anti-malarial drugs are categorized in blood schizonticides, tissue schizonticides, hypnozoiticides and transmission-blocking drugs (gametocytocides and sporontocides) [46, 47]. Blood schizonticides inhibit the development of asexual erythrocyte forms of the *Plasmodium* parasites while tissue schizonticides prevent the relapse of *P. ovale* and *P. vivax* hypnozoites in the liver stage. On the other hand, gametocytocides destroy female and male gametocytes of the parasites thereby inhibiting the transfer of malaria from an infected person to uninfected female *Anopheles* mosquito. For their part, sporontocides disrupt oocysts found in the mosquito stage of the *Plasmodium* life cycle, thus hampering the transmission of the disease [47]. Below, the most commonly used anti-malarial drugs are presented and their distinct pharmacological properties briefly described.

The first successful anti-malarial drug used was quinine. It acts as a blood schizonticide against all human malaria parasite species and as gametocytocide for only *P. vivax*, *P. ovale* and *P. malariae* [48]. Due to its reported resistance in most malaria-endemic regions, oral quinine is currently recommended in combination with antibiotics (tetracycline, doxycycline, azythromycin or clindamycin) in the treatment of uncomplicated malaria [2, 49]. However, intravenous injection of quinine injectable is one of the effective options to date to treat severe malaria [2].

Today, artemisinin derivatives (e.g. dihydroartemisinin, artemether, artesunate) are the most important compounds in the therapeutic arsenal against malaria. They have potent and rapid activity against asexual parasites of all *Plasmodium* species, killing all stages from young rings to schizonts. They also eliminate the gametocytes in *P. falciparum* malaria [2, 50]. To delay the emergence of resistance to the artemisinin derivatives, the WHO recommends to all the endemic countries to switch their first-line treatments against uncomplicated malaria to artemisinin-based combination therapy (ACT) [2, 51]. Indeed, since 2010, five artemisinin-based combinations have been registered by the WHO, namely artemether-lumefantrine, artesunate-amodiaquine, dihydroartemisinin-piperaquine, artesunate-mefloquine and artesunate-sulfadoxine/pyrimethamine [2, 51, 52]. More recently, artesunate-pyronaridine, another fixed-dose ACT, was included in the WHO list of prequalified medicines for malaria [53, 54]. To fight against reported recrudescence and treatment failures in Africa and Asia [4, 55], artemisinin-based combinations are increasingly combined with primaquine or antibiotics (azithromycin, clindamycin or doxycycline) [56]. Clinical efficacies of triple ACT are also investigated [57]. Based on recent trials, intravenous or intramuscular artesunate injectable is currently one of the treatments of choice against severe malaria [1, 2].

Halofantrine and lumefantrine that belong to the arylamino alcohol pharmacophore-type like quinine also act as schizonticide [58]. However, the usefulness of halofantrine as an anti-malarial is currently limited due to its cardiotoxicity [59, 60]. On the other hand, lumefantrine is only administered against uncomplicated falciparum and vivax malaria in combination with artemether [2]. Mefloquine, another quinine derivative is commonly used as prophylaxis of *Plasmodium* malaria [58]. Its combination with artesunate is recommended for the treatment of falciparum malaria in Southeast Asia while, in Africa, the other artemisinin-based combinations are preferred because of the emergence of mefloquine resistance in this part of the world [2].

Chloroquine, a rapid schizonticide with gametocytocidal properties, has been the gold standard for the prophylaxis and treatment of malaria in the last half century due to its affordability and efficacy [61]. Today, the drug is less effective due to the widespread emergence of *P. falciparum* resistant strains [61, 62]. Yet, chloroquine continues to be used for the treatment of uncomplicated malaria caused by *P. vivax*, *P. malariae* and *P. ovale* [63]. Amodiaquine is an anti-malarial drug that faces several limitations due to cross-resistance with chloroquine because of their structural similarity [64]. Nevertheless, as mentioned above, amodiaquine is widely used in combination with artesunate in many endemic countries [2]. On the other hand, piperaquine, a drug that has also structure similarity with chloroquine, possesses rapid parasite-clearing activity [63]. However, its monotherapy is associated with the survival of gametocytes in the peripheral blood, which may facilitate the spread of resistant parasites [65]. This is why piperaquine is combined with dihydroartemisinin, a strong gametocytocide drug in malaria [2].

Effective against intrahepatic forms of all *Plasmodium* species, primaquine is used as chemoprophylactic drugs for all types of malaria [2, 66]. According to WHO recommendations, a 14-day regimen of primaquine is required to treat *P. vivax* and *P. ovale* hypnozoites (and prevent the appearance of erythrocytic forms of the parasite), which is a challenge in terms of treatment compliance [19, 67]. Primaquine is also a sporontocidal and gametocytocidal drug [47, 67]. Nowadays, a single-dose treatment of tafenoquine is also known to be effective against intrahepatic forms of malaria [68]. However, these two drugs can trigger an acute haemolytic anaemia in patients with glucose-6 phosphate dehydrogenase deficiency [19, 69, 70]. Unfortunately, this vulnerable group of patients represents up to 35% of people in countries affected by *P. vivax* and *P. ovale* malaria [67]. When administered in combination with chloroquine or ACT, primaquine can provide high effective relapse prevention and radical cure of *P. vivax*, *P. ovale* and *P. falciparum* malaria [2, 71]. For its part, atovaquone also inhibits pre-erythrocytic development (in the liver) and oocyst development (in the mosquito) of all *Plasmodium* species [72, 73]. Due to monotherapy resistance [74], it is usually co-administered with proguanil for the prophylaxis of malaria and the treatment of chloroquine-resistance malaria [73, 75].

The combination of sulfadoxine and pyrimethamine has been used for many years to treat uncomplicated and chloroquine-resistant *P. falciparum* malaria. This association is currently recommended as intermittent prevention treatment for pregnant women (and infants) in most malaria-endemic African countries [1, 2, 76, 77]. They are also indicated in the malaria prophylaxis of travellers in areas where they still remain effective [78]. Sulfadoxine + pyrimethamine can be combined with artesunate or amodiaquine to reduce treatment failure in uncomplicated malaria in chloroquine-resistant regions [1, 2, 79]. The intermittent administration of amodiaquine plus sulfadoxine-pyrimethamine to children is also recommended as seasonal malaria chemoprevention in some countries [30].

Due to delayed parasite clearance, high treatment failure rates and *Plasmodium* resistance observed with most of existing drug regimens (monotherapies and combination therapies) [3, 5, 6], many efforts are done to discover or develop new and effective anti-malarial agents from plant, marine or (bio)synthetic sources [80, 81]. Among the drugs that are currently under development (e.g. preclinical research and clinical development), one can cite cryptolepine and curcumin, two plant-derived products, and their semi-synthetic analogues that are useful for combating various types of *Plasmodium* species [82–88]. This is also the case of fosmidomycin, an antibacterial agent that demonstrated high efficacy in the treatment of uncomplicated *P. falciparum* malaria when used in combination with clindamycin, piperaquine or artesunate [46, 89–91].

Of note, current research in drug discovery are also focused on the synthesis of others artemisinin derivatives (e.g. artemisone, artemiside) and artemisinin-related agents that are more potent, more bioavailable, hydrolytically stable, and less toxic than available artemisinin-type compounds [92–95]. The synthesis of anti-malarial hybrid compounds that hybridized different types of pharmacophores is also the current focus of many scientists. This category includes artemisinin-, quinoline- and ferrocene-based hybrid compounds [47]. Finally, different fatty acids isolated from marine plants and sponges have shown enormous potentialities as liver schizonts-directed anti-malarials [96, 97].

## Liposomes as pharmaceutical carriers for the prevention and treatment of malaria

**Fig. 2:**
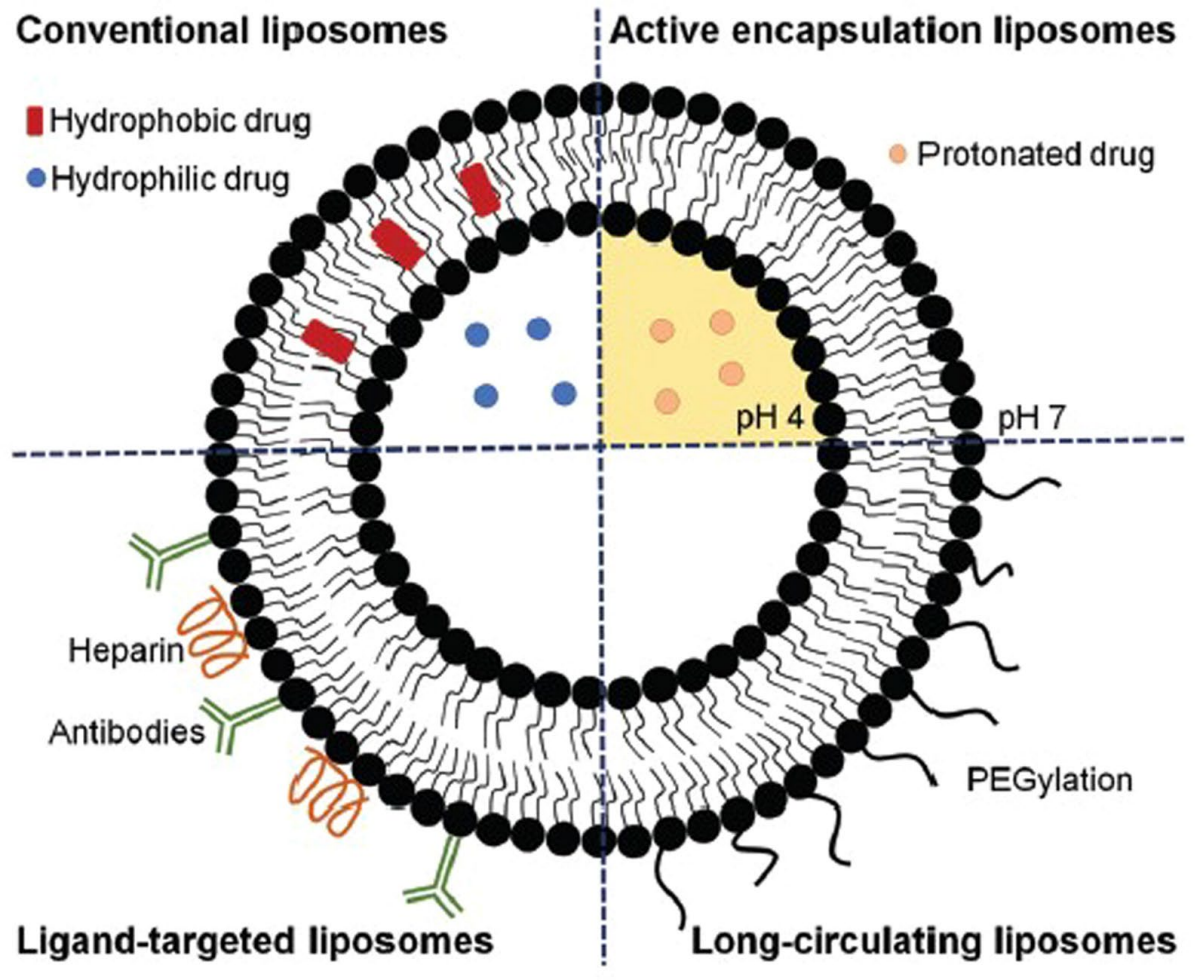
Schematic representation of liposomes developed using different strategies aiming at improving the therapeutic efficacy of antimalarial drugs and antigens. Image from reference [8], reprinted with permission from the Royal Society of Chemistry

Liposomes are small (30 nm to several micrometres) and artificial spherical vesicles that contain one or several phospholipid bilayers enclosing an aqueous core (Fig. 2) [98]. On the basis of their size and number of bilayers, liposomes are classified into multilamellar vesicles (MLV) (>500 nm), oligolamellar vesicles (OLV) (100–500 nm), giant unilamellar vesicles (GUV) (>1000 nm), large unilamellar vesicles (LUV) (100–1000 nm), and small unilamellar vesicles (SUV) (10–100 nm) [98–101].

The rigidity, permeability, and charge of these bilayers depend on their lipid composition. The major components of liposomes are natural or synthetic phospholipids (55–100%) and cholesterol (30–45%) [101].

Among the lipids frequently used in the formulation of liposomes, one can cite soybean phosphatidylcholine (SPC), egg phosphatidylcholine (EPC), dimyristoylphosphatidylcholine (DMPC), dipalmitoylphosphatidylcholine (DPPC), distearoylphosphatidylcholine (DSPC), dibehenoylphosphatidylcholine (DBPC), dipalmitoylphosphatidylglycerol (DPPG), dimyristoylphosphatidylglycerol (DMPG), distearoylglycerophosphoethanolamine (DSPE), and cholesterol [99, 101, 102]. Cholesterol is added in the liposomal formulations for providing rigidity, elasticity and permeability on membrane bilayers and therefore better stability to the liposomes. The molar percentage of cholesterol depends on the sought-after physico-chemical characteristics [99, 101].

Due to their dual hydrophilic and hydrophobic nature, biodegradability, biocompatibility and low toxicity, liposomes represent a potentially interesting platform for the (co-)delivery of water-soluble and lipophilic anti-malarial drugs [10, 98, 99]. Moreover, in the early 1980s, Gregoriadis demonstrated that liposomes coated with cell specific ligands may prove highly useful as carriers for site-specific delivery of drugs in the biophase [103]. Indeed, their site-specific drug delivery potential makes them a useful delivery system for targeting infected hepatocytes and RBCs viz*.*, passive and active targeting. Passive drug targeting is achieved by the use of conventional liposomes and long-circulating liposomes (PEGylated liposomes). In contrary, active drug targeting is realized by the surface activation of the liposomes through inclusion of surface ligands (e.g. glycolipids, carbohydrate, peptides, proteins or antibodies) binding to the infected red blood cells (RBCs) associated receptors [7, 8, 10].

To prepare liposomes, literature reports different methods of preparation (e.g. thin lipid film hydration and reverse-phase evaporation), drug encapsulation (e.g. passive loading and active loading), reduction and homogenization of size (e.g. membrane extrusion and sonication). For more details, the reader can refer to more specialized reviews [98–101, 104].

### Liposome as passive targeting drug delivery system for malaria therapy

#### Liposomes for the treatment of liver stage of malaria

Targeting hepatic stage of malaria by liposome drug delivery systems constitutes an attractive and promising approach to treat pre-erythrocytic forms of malaria and prevent the progression of the disease to the erythrocytic stages that are responsible for clinical symptoms. In fact, after oral or parenteral delivery, liposome-based nanocarriers are naturally captured by the cells of the mononuclear phagocytic system (MPS), also known as the reticuloendothelial system (RES). Indeed, about 70–80% of liposomal nanocarriers are trapped in the Kupffer cells (also known as stellate sinusoidal macrophages) of the liver and 5–8% are engulfed by the macrophages residing in the spleen. These two types of cells (stellate and splenic macrophages) belong to the MPS [105–107].

Selective targeting of liposomes to the hepatocytes in the liver, but not to Kupffer cells, can be achieved by adjusting their lipid compositions and their physicochemical properties (e.g. size and zeta potential). Indeed, small liposomes are able to pass through the ~ 100 nm pores of the fenestrated endothelium lining the hepatic sinusoid followed by interaction with and internalization by the hepatocytes [108, 109]. In contrast, large liposomes were taken up by the Kupffer cells, but not by the hepatocytes, in the liver [108, 110]. Thus, encapsulating anti-malarial drugs in small liposomes makes an intriguing strategy for targeted delivery to the hepatocytes thereby enhancing their therapeutic efficacy. Unfortunately, small liposomes are characterized by low encapsulation volumes, leading to low drug loading capacities, as often observed when using passive loading procedures [108]. Therefore, active or remote loading strategies constitute an interesting alternative, allowing the liposomes to achieve drug encapsulation efficiencies close to 100%. In these procedures, pH gradient represent the driving force that can be established by changing the pH of the extraliposomal phase [98, 100, 108].

To prevent opsonization and further clearance of liposomes by the MPS, their surface properties are often altered by PEGylation, a phenomenon that consists of conjugating covalently the surface of nanocarriers with polyethylene glycol (PEG). In other words, PEGylation enhances the circulation or retention time of liposomes in the blood, which can be a prerequisite in the case of malaria.

The use of liposomes as carriers for drug delivery was introduced in the 1970s [111, 112]. However, their application in the chemotherapy of murine malaria took place a few years later. Indeed, in 1979, Pirson et al*.* encapsulated for the first time primaquine in multilamellar and negatively charged cholesterol-rich liposomes and evaluated their anti-malarial activity in *Plasmodium berghei*-infected mice [113]. The composition of these developed liposomes is shown in Table 1.

**Table 1:** Composition of liposomes used as passive targeting drug delivery systems in malaria therapy

Drugs	Lipid composition	Ratio^a^	References
Primaquine	EPC: PS: cholesterol	4:1:5	[110, 113–116]
Chloroquine	Fluid-state		[117–120]
	PC: PG: cholesterol	10:1:5	
	Gel-state		
	DSPC: DPPG: cholesterol	10:1:5 10:1:10	
Primaquine Leu-primaquine Ala-leu-primaquine Ala-leu-ala-leu-primaquine	Fluid-state		[121]
	CHEMS: cholesterol: PL100	1:4:10	
	CHEMS: cholesterol: PL90G	1:4:10	
	CHEMS: cholesterol: PL100	1:2:7	
	CHEMS: cholesterol: PL90G	1:2:7	
	CHEMS: PL100	1:10	
	CHEMS: PL90G	1:10	
	CHEMS: PL100	1:7	
	CHEMS: PL90G	1:7	
	Gel-state		
	CHEMS: cholesterol: PL100H	1:4:10	
	CHEMS: cholesterol: DPPC	1:4:10	
	CHEMS: cholesterol: PL100H	1:2:7	
	CHEMS: cholesterol: DPPC	1:2:7	
	CHEMS: PL100H	1:10	
	CHEMS: DPPC	1:10	
	CHEMS: PL100H	1:7	
	CHEMS: DPPC	1:7	
Arteether	DMPC DMPC: DPPC DMPC: DPPC: cholesterol EPC EPC: cholesterol EPC: cholesterol DPPC DPPC: DBPC: cholesterol DPPC: DBPC: cholesterol DPPC: DBPC: cholesterol DPPC: DBPC: cholesterol	1 1:1 1:1:1 1 1:0.5 1:1 1 1:1:1 1:1:2 1:1:2 0:1:1	[122]
Arteether	DPPC: DBPC: cholesterol	1:1:2	[123]
Desferrioxamine	EPC: EPG^c^ EPC: EPG: cholesterol^d^ DSPC: DPPG: cholesterol^e^	10:1 10:1:4 10:1:4	[124]
β-artemether	EPC: cholesterol	1:0 1:1 2:1 3:1 4:1 3:2 4:3	[125]
Artesunate	EPC: cholesterol	4:3	[126]
Chloroquine	SPC: cholesterol	10:0 10:1 8:1 5:1 4:1 3:1	[127]
Artemisinin Artemisinin + Curcumin	PL90G: cholesterol	5:0.6 1.6:0.2	[128, 129]
	PL90G: cholesterol: DSPE-mPEG2000	5:0.6:0.25 1.6:0.2:0.1	
–	SPC: phophatidic acid SPC: stearylamine SPC: stearic acid SPC: stearyl alcohol SPC: myristic acid	80:20	[130]
	SPC: Stearylamine: cholesterol: DSPE-mPEG2000	80:20 ^b^	
Curcumin	SPC	100	[131]
β-methasone hemisuccinate	HSPC: cholesterol: DSPE-PEG2000	55:40:5	[132, 133]
Monensin Doxycycline	SPC: cholesterol SPC: cholesterol: stearylamine SPC: cholesterol: DSPE-mPEG2000 SPC: cholesterol: stearylamine: DSPE-mPEG2000	7:3 7:3 (± 10 mol %) 7:3 (± 0.5–5 mol %) 7:3 (± 10 mol % ± 0.5–5 mol %)	[134, 135]
Indocyanin Green	PL S75: cholesterol PL S75: cholesterol: DSPE-mPEG2000	9:1 (w/w) 9:1:1.33 (w/w)	[136]
Platinum-chloroquine diphosphate dichloride	HSPC: cholesterol: DSPE-mPEG2000: DOTAP	55:45:5:0 50:45:5:5 45:45:5:10 40:45:5:15 35:45:5:20	[137]
Maduramicin	SPC: cholesterol (± DSPE-mPEG2000)	7:3 (± 5 mol %)	[138]
Primaquine + Chloroquine	HSPC: cholesterol: DSPE-mPEG2000	55:45:5	[139]

^a^Unless otherwise indicated, the ratio is expressed in molar

^b^The 20 parts are made up of 0–20 mol % of SA, 0, 5,10, 15 or 20 mol % of cholesterol, and 0, 1, 2.5 or 5 mol % of DSPE-mPEG2000

^c^Low bilayer-rigidity liposomes

^d^Intermediate bilayer-rigidity liposomes

^e^High bilayer-rigidity liposomes

Following a single intravenous injection (60 mg/kg), primaquine-loaded liposomes showed a potent anti-malarial activity leading to 100% cure in treated mice [113, 114]. The anti-malarial activity of this liposomal formulation was associated with a reduced subacute toxicity compared with free primaquine, which exhibited severe drug toxicity at 30 mg/kg [113–115]. Administered at dose corresponding to primaquine 25 mg/kg, primaquine-entrapped liposomes and free primaquine cured 75% and 55% of infected mice, respectively [113]. This liposomal primaquine-based regimen was also accompanied with a selective accumulation of the drug in reticulo-endothelial cell-rich tissues (e.g. liver and spleen) and a reduced access to non-target tissues (e.g. lungs, heart, kidneys, brain, pancreas and muscle tissues) [115, 116]. Moreover, primaquine encapsulated in these liposomes was eliminated more slowly than its free drug molecules counterparts [115, 116]. To confirm these observations, Smith et al*.* [110] evaluated the uptake and distribution of liposomal primaquine in isolated perfused rat liver. The results indicated that the uptake of the primaquine-loaded liposomes was gradual reaching a plateau after 20 min of perfusion, with a hepatic uptake of ca. 60% of the initial load. In contrast, clearance of free primaquine reached a plateau at 44% of the initial load within 5 min confirming radical differences in the intrahepatic distribution of free and entrapped liposomes [110].

In another study, the influence of composition (or rigidity) of liposomal membrane bilayers on tissue distribution of primaquine was evaluated in mice [121]. The composition of tested vesicular systems is summarized in Table 1. Data indicated that the accumulation of gel-state primaquine liposomes in the liver and spleen is greater than that of liquid-crystalline state liposomes. Additionally, primaquine entrapped with gel-state liposomes composed of Phospholipon-100 H (PL-100H, hydrogenated or saturated SPC), cholesteryl hemisuccinate (CHEMS) and cholesterol (10:1:4, molar ratio) prevented more non-target tissues from accumulation than others gel-state liposomes [121].

#### Liposomes for the treatment of erythrocytic stage of malaria

Passive targeting intra-erythrocytic stage of malaria attracted immense attention of researchers due to their great potential for the treatment of malaria. Indeed, liposomes loaded with anti-malarial compounds may offer better therapeutic outcomes and promise to be an efficacious option adaptable for clinical application.

Crommelin and colleagues were among the first researchers to assess the schizontocidal efficacy of liposomes containing anti-malarial compounds [117–120]. In their investigations, they confirmed that the rigidity of liposomal bilayer has an effect on the pharmacological activity and the toxicity of liposomes containing anti-malarial drugs (see composition in Table 1). Indeed, the total blood concentrations of intraperitoneal injections of chloroquine encapsulated in gel-state liposomes were significantly higher than that of chloroquine loaded in fluid-state liposomes [117, 118]. As a result, gel-state liposomes containing chloroquine (2–6 mg per mouse, day 1–5 post-infection or day 1, 2 and 3 prior to infection, intraperitoneal) increased considerably its capacity to prevent and treat a *P. berghei* infection in mice compared to fluid-state liposomes containing chloroquine at the same dose [118]. Similar results were obtained when chloroquine loaded in fluid-state liposomes were intraperitoneally administered to *P. berghei*-infected mice on day 7–10 post-infection [119]. Nevertheless, the liposomal delivery of chloroquine using these gel and fluid state liposomes overcame drug resistance in *P. berghei*-infected mice in comparaison to free chloroquine [117, 118, 140]. Noteworthy, intramuscular or subcutaneous injection of both fluid- and gel-state liposomes containing chloroquine (8 mg/mouse) led to 100% protection of *P. berghei*-infected mice for at least 10 days [120]. This remarkable efficacy was attributed to the sustained release of chloroquine from the site of administration as confirmed by preliminary pharmacokinetic data [120].

The gel-state liposomes were made up of DSPC, DPPG, and cholesterol, as shown in Table 1. Used together, DSPC, a neutral phospholipid, and DPPG, a negatively charged phospholipid achieved a high encapsulation efficiency of chloroquine by passive loading method [118, 127]. In fact, at pH 7.4, the positively charged chloroquine easily interacts with the negatively charged liposomes, which is important in the passive loading technique [127]. However, DSPC and DPPG are more expensive than natural and neutral phospholipids such as SPC and EPC. This constitutes a weakness from an industrial and economic point of view. This is the reason why Qiu et al*.* [127] developed chloroquine-loaded liposomes composed of SPC or EPC. To achieve enhanced encapsulation efficiency of chloroquine, a transmembrane pH-gradient method was performed. The results were in line with those obtained previously with primaquine, quinine and chloroquine [108, 141]. Noteworthy, these three anti-malarial agents are polyprotic drugs that exhibit multiple ionization states and different degree of lipophilicity as a function of physiological conditions. In this context, Fernàndez-Busquets and colleagues designed distribution models that precisely describe the partitioning behaviour of these drugs in liposome vesicular systems [142]. These models have the capacity to predict the interactions of polyprotic drugs with biological and synthetic membranes depending on the pH, lipid composition and phospholipid charge. Importantly, these anti-malarial drug distribution modelling improved the effective drug delivery strategies such as liposome-based active drug encapsulation methods driven by transmembrane pH gradients [142].

Al-Angary et al*.* [122] prepared a series of arteether liposomal formulations that contained different phospholipids (e.g. DMPC, DPPC, EPC and/or DBPC) with or without cholesterol (see Table 1 for the composition). Thereafter, they evaluated the in vitro release rate of the drug from the liposomal systems. Characterized by a fast phase (for 2 days) followed by a slower phase (for about 18 days), this release rate was 0.94–1.20% per day for EPC, DPPC and DMPC liposomes. These values were higher than that of arteether loaded in liposomal systems containing phospholipids of long acyl chain length with or without cholesterol. However, it should be noted that liposomes containing EPC, of higher molecular weight and having unsaturated fatty acids, exhibited relatively low trapping efficiency due to the higher ability of arteether to be loosely adsorbed at the phospholipid/water bilayer interface.

In a follow-up study, arteether loaded in liposomes that consisted of DPPC, DBPC and cholesterol (molar ratio of 1:1:2) was selected for in vivo evaluation [123]. This arteether liposomal formulation was administered orally and intravenously to rabbits at a dose of 50 mg/kg. The absolute bioavailability was approximately 98% for the oral arteether liposomes compared with 32% for the oral arteether aqueous suspension. Moreover, the rate of absorption of arteether from the liposome vesicles was faster than that from the aqueous suspension. Indeed, T_max_ of oral arteether liposomes and of oral aqueous suspension were 47.5 min and 117 min, respectively. On the other hand, the extent of arteether absorption was higher in the case of oral liposome formulation (C_max_ = 3.03 µg/ml) compared with the oral aqueous suspension (C_max_ = 0.55 µg/ml).

The use of liposomes as carriers for other artemisinin derivatives (artemether and artesunate) was also assayed by Plaizer-Vercammen and colleagues [125, 126]. In these studies, a series of liposomes were prepared using EPC and cholesterol at different ratios, as shown in Table 1. The selected liposome formulations contained 300 mg of lipids (EPC:cholesterol; 4:3, w/w) and 1.5 mg of artemether or artesunate. Interestingly, a dialysis test demonstrated that more than 80% of both artemether and artesunate were released from these liposome formulations, reaching equilibrium within 24 h. Administered intravenously, the multilamellar vesicles containing artemether (4.8 mg/kg, day 3 and 4 post-infection, intraperitoneal injection) cured 100% of mice infected with *Plasmodium chabaudi chabaudi*, a virulent rodent malaria parasite.

Furthermore, Isacchi et al*.* [128] proposed the encapsulation of artemisinin in both conventional and long-circulating liposomes in order to improve its biopharmaceutical properties. As summarized in Table 1, artemisinin-loaded in conventional liposomes were prepared using Phospholipon 90G (P90G, soybean lecithin at ≥ 90% of phosphatidylcholine) and cholesterol as lipid phase while the long-circulating liposomes were obtained by mixing this lipid phase with DSPE-mPEG2000. The data revealed that, after intraperitoneal administration in mice, encapsulation of artemisinin into liposomes prolonged its circulating time in the blood. Nevertheless, the PEGylated liposomes appeared to have a better pharmacokinetic profile.

Additionally, these nanoencapsulated artemisinin formulations resulted in less variability in the plasma concentrations of the drug compared with free artemisinin, which showed a fluctuant trend in its blood concentration [129]. As a consequence, artemisinin loaded in both conventional and PEG-containing liposomes exhibited, at 50 mg/kg/day × 12 days, an immediate and constant anti-malarial effect in *P. berghei* infected mice. The period of survival exceeded 30 days post-infection for all the mice treated with liposomal formulations. Nevertheless, this therapeutic effect was more pronounced with artemisinin PEGylated liposomes. By contrast, free artemisinin that showed a fluctuant blood concentration in mice began to decrease the parasitaemia levels only 7 days after the start of treatment. This finding was reflected in the low anti-malarial effectiveness of free artemisinin. In combination with curcumin loaded-liposomes (100 mg/kg/day × 12 days, intraperitoneal), artemisinin liposomal vesicles cured all the malaria-infected mice.

In another study, Aditya et al*.* [131] developed SPC-based liposomes for the encapsulation of curcumin (see Table 1). After intravenous administration in *P. berghei*-infected mice, this formulation exhibited much more delay in parasitaemia progression when compared to control group (untreated mice). However, it prolonged survival of the treated mice only up to 11 days. Hence, these curcumin-loaded liposomes (40 mg/kg) were combined with an intramuscular oily solution of α/β-arteether (30 mg/kg). The results indicated that this combination therapy was able not only to cure *P. berghei*-infected mice but also to prevent recrudescence (100% survival and mean survival time >50 days).

Di-ART-GPC conjugate is a potent anti-malarial drug synthesized by a facile esterification of artesunate (ART) and glycerophosphorylcholine (GPC) as a linker [143]. After self-assembly approaches (without excipient), Di-ART-GPC formed multilamellar liposomes with high drug loading (~ 80%) [143]. The in vitro antiplasmodial activity of the assembled Di-ART-GPC liposomes (IC_50_ 0.39 nM) against *P. falciparum* 3D7 strain was superior to that of the conjugate (IC_50_ 1.90 nM) and the free artesunate (IC_50_ 3.13 nM) tested separately. The novel amphiphilic dimeric artesunate phospholipid conjugate-based liposomes were administered to *P. berghei*-infected mice through intravenous injection considering equivalent artesunate dosages of 15, 30 and 60 mg/kg for 4 consecutive days. According to the data, the formulated liposomes exhibited much longer retention half-life leading to far better parasites killing and delayed recrudescence as well as improved survivability, in comparison with the free artesunate [143].

Desferrioxamine, a hydrophilic iron chelator, was proposed in the treatment of malaria due to its capacity to inhibit parasite growth in vitro and parasitaemia in vivo [144–148]. However, this drug presents a poor oral bioavailability as well as a short-life (5–10 min) after intravenous injection thereby requiring continuous administration for optimal effectiveness [147, 149]. To overcome these drawbacks, Postma et al*.* [124] developed liposomal carrier systems for sustained release of desferrioxamne after subcutaneous injections. The composition of these liposome formulations is presented in Table 1. Administered in prophylaxis two subsequent days before *P. berghei* infection in mice, desferrioxamine liposomes (200–1000 mg/kg/day) suppressed parasitaemia on day 8 post-infection in a dose-dependent way. By contrast, free desferrioxamine administered over the same treatment schedule did not suppress parasitaemia within the equivalent post-inoculation period of time. In fact, parasitaemia in the group of mice treated with the free drug enhanced similarly than that of group of untreated mice [124].

Ghosh and co-workers evaluated the in vitro antiplasmodial effect of stearylamine loaded in SPC-liposomes [130]. Interestingly, a high inhibition of growth and multiplication of *P. falciparum* were obtained at IC_50_ value of 6.87 µM. Incorporation of either cholesterol or DSPE-mPEG2000 in stearylamine-SPC liposomes further improved this antiplasmodial activity. On the other hand, stearylamine loaded in EPC-liposomes also exhibited an effective antiplasmodial activity (IC_50_ = 7.30 µM). However, up to concentrations of 60 µM, stearylamine loaded in sphingomyelin-liposomes had no effect on growth of parasites. In addition, blank-SPC liposomes, phosphatic acid-SPC liposomes, stearic acid-SPC liposomes, stearyl alcohol-SPC liposomes and palmitic acid-SPC liposomes were unable to elicit any antiplasmodial activity [130]. The composition of these different liposome delivery systems are summarized in Table 1.

All these data indicated that the inhibition of parasite growth was regulated by the chain length of the alkyl group, the density of stearylamine and the presence of PEG functionalized lipids in the liposomes. This also confirmed that the interaction of liposomes with mammalian cells is significantly dependent on the composition, the charge, the fluidity and the hydrophilicity on the surface of the liposomes [130].

In a follow-up study [134], Ghosh and colleagues developed SPC-cholesterol, stearylamine-SPC-cholesterol liposomes and phosphatic acid-SPC-cholesterol (without or with different densities of DSPE-mPEG2000) containing monensin, a polyether antibiotic ionophore (see Table 1 for the composition). It was found that, in vitro, stearylamine-SPC-cholesterol liposomes containing monensin (IC_50_ 0.74 nM) were 4.2-fold more active against *P. falciparum* 3D7 than the free drug (IC_50_ 3.17 nM). On the other hand, monensin SPC-cholesterol and monensin phosphatic acid-SPC-cholesterol showed IC_50_ values of 1.11 nM and 2.98 nM, respectively. After incorporation of different densities of DSPE-mPEG2000 (0.5, 2.5 and 5 mol%) on the surface of SPC-cholesterol and phosphatic acid-SPC-cholesterol, the in vitro antiplasmodial activity of monensin was significantly enhanced in comparison with conventional forms of liposomes. However, no observable difference on the growth of parasites was observed with monensin loaded in positively charged stearylamine-SPC-cholesterol liposomes grafted with different densities of PEG [134]. Administered in mice infected with *P. berghei*, monensin liposomes (8 mg/kg × 4 days) from different chain lengths (750, 1000, 2000, 3000 and 5000) of DSPE-mPEG (2.5 mol%) bearing SPC-cholesterol modulated the circulation life of the encapsulated drug thereby leading to an important reduction of parasitaemia and improved survival times (20.5–24.5 days) compared with the untreated group of mice (15.5 days). Nevertheless, the results indicated that liposomes made of DSPE-mPEG2000 exhibited higher anti-malarial efficacy than those composed of DSPE-PEG750, 1000, 3000 and 5000 [134]. The authors also investigated the in vivo anti-malarial efficacy of monensin (8 mg/kg × 4 days) intercalated in liposomal formulations containing different densities of PEG2000 (0.5, 2.5 and 5 mol%). Data indicated that monensin loaded in stearylamine-SPC-cholesterol liposomes with 5 mol% of PEG was the most effective (mean survival time = 27.5 days) followed by SPC-cholesterol liposomes with 5 mol% of PEG (mean survival time = 23.5 days). All these above-mentioned in vitro and in vivo results clearly demonstrated that the inhibition of growth of *Plasmodium* parasites depends on the lipid composition of liposomes as well as the densities and chain length of DSPE-mPEG on their surface. Nevertheless, a more pronounced anti-malarial efficacy of monensin (6 mg/kg) loaded in stearylamine-cholesterol-DSPE-mPEG2000 (5 mg/kg) liposomes in combination with free artemisinin (40 mg/kg) was observed in mice infected with two different strains of *P. berghei.* Indeed, this co-administration eliminated completely the parasite burden leading to a 100% survival rate [134].

Following their previous studies, Ghosh and co-investigators intercalated doxycycline into both conventional and long circulating PEGylated stearylamine-SPC liposomes (see the composition in Table 1) [135]. After 48 h of incubation in a *P. falciparum *in vitro culture, doxycycline stearylamine-SPC-cholesterol liposomes and doxycycline SPC-cholesterol liposomes exhibited IC_50_ values of 0.36 µM and 0.85 µM, respectively, suggesting a marked growth inhibition of parasites compared with the free drug (IC_50_, 14 µM). These results were in line with those previously reported [134]. In mice infected with either chloroquine-sensitive or a chloroquine-resistant *P. berghei* strain, the PEGylated liposomal formulations containing doxycycline (2.5 mg/kg × 4 days, subcutaneous injection) decrease the burden of blood parasite from about 40% to less than 0.2%. In contrast, free doxycycline administered subcutaneously at 2.5 mg/kg for 4 days showed negligible clearance in both strain of *P. berghei*. The reduction of parasitaemia in group of mice treated with doxycycline-loaded PEG liposomes was reflected by their mean survival time (> 40 days), which is threefold higher than that of mice treated with free doxycycline (<14 days). On the other hand, the placebo formulations (without stearylamine and doxycycline) had no observable effect on the killing of parasites [135].

Like monensin, maduramicin is a carboxylic ionophore that demonstrated the potential to rapidly eliminate asexual *P. falciparum* parasites and prevent the transmission of malaria infection in mice model by hindering gametocyte, sporogony and oocyst development [150, 151]. However, despite the potent anti-gametocytocidal properties of maduramicin, its application in chemotherapy is restricted due to its high lipophilic nature and toxicity, affecting the heart and skeletal muscles [138, 152]. To alleviate these weaknesses, Ghosh and colleagues developed conventional and PEGylated liposomes containing maduramicin (see composition in Table 1) [138]. Interestingly, maduramicin in PEGylated liposomes displayed enhanced antiplasmodial activity in vitro (IC_50_ = 1.25 ng/ml and 1.20 ng/ml against chloroquine sensitive and chloroquine resistant strains of *P. falciparum*, respectively), much better than free maduramicin (IC_50_ = 2.5 ng/ml and 2.8 ng/ml against chloroquine sensitive and resistant *P. falciparum* strains, respectively) [138]. After treatment with the PEGylated liposomes, no obvious toxic effects were observed on vital organs such as kidney and liver. The inhibitory action of conventional liposomal formulations of maduramicin on the selected *Plasmodium* strains was found to be similar to that of free maduramicin. Additionally, the authors observed complete clearance of parasite load (~ 0% parasitaemia) from blood after subcutaneous administration of PEGylated liposomal maduramicin (1.5 mg/kg for 4 consecutive days, 48 h post-infection) in the chloroquine-resistant *P. berghei*-infected mouse model (*versus* 13% and 49% parasitaemia in free maduramicin-treated mice and untreated mice, respectively). Consequently, all the mice treated with maduramicin-loaded PEGylated liposomes showed a median survival time of > 45 days (without any relapse of the infection thereby preventing the emergence of drug resistant parasites). In contrast, the untreated group of mice displayed a median survival time of 17 days while animals treated with free maduramicin showed a median survival time of 29 days [138]. Furthermore, the PEGylated liposomal formulation of maduramicin (4 mg/kg, single dose, subcutaneous) showed substantial prophylactic activity in mice infected with *P. berghei*. The enhanced efficacy of maduramicin-loaded PEGylated liposomes may be due to its stealth properties: e.g. improved circulatory half-life, enhanced uptake by infected RBCs, protection from phagocytosis, opsonization and degradation [138].

Trans platinum-chloroquine complex (PtCQ) is a new type of anti-malarial drugs synthesized by mixing platinum and chloroquine diphosphate [137]. To increase the bioavailability of this poorly-water complex that shows therapeutic effects against *Plasmodium* parasites resistant to traditional drugs, Ibrahim et al*.* [137] encapsulated PtCQ into PEGylated neutral and cationic liposomes. Neutral PtCQ-loaded liposomes were prepared using hydrogenated SPC (HSPC), cholesterol and DSPE-mPEG2000 at the molar ratio of 55:45:5. On the other hand, cationic PtCQ-loaded liposomes were composed of HSPC, cholesterol and DSPE-mPEG2000 in which 1,2-dioleoyl-3-trimethylammoniumpropane (DOTAP), a cationic lipid was incorporated at different ratios (0, 5, 10, 15 and 20 mol%) (see Table 1). Indeed, the ability of cationic liposomes to be adsorbed onto the surface of erythrocytes and to fuse with the cellular membranes of these cells is enhanced with the exposure of anionic lipids (e.g. phosphatidylserine) located within the outer surface of erythrocytes. This exposure results in the scrambling of lipids located on the inner monolayer of erythrocytes during their suicidal death by the malaria parasites (also known as eryptosis) [137, 153–155]. The drug release studies indicated that, at 0 h, all the PtCQ liposomes loaded by thin drug-lipid film method (drug to lipid ratio of 1:7, w/w) showed significant burst release (8.9 to 13.2%). After 72 h, cationic PtCQ liposomes containing 5 mol% of DOTAP and neutral PtCQ liposomes released 25.8% and 17.8% of drug, respectively. The higher drug release of cationic liposomes may be due to electrostatic repulsion between the cationic lipid and the cationic drug [137]. On the contrary, cationic PtCQ liposomes containing 5 mol% of DOTAP and loaded using an ammonium sulfate gradient (drug to lipid ratio of 1:7, w/w) showed sustainably drug release (~ 4%) without initial burst release, thus showing that these formulations may provide controlled release of the encapsulated drug. Importantly, no significant effect on the drug release was observed after increasing the molar ratio of DOTAP (10–20 mol%) suggesting that the surface charge has no effect on the release of chloroquine loaded using ammonium sulfate and pH gradients [137].

As stated above, chloroquine can be combined with a single dose of primaquine to provide prophylactic therapy and radical cure for erythrocytic phase infection of *Plasmodium* parasites, as well as high effective treatment of acute infections caused by sporozoites and/or malaria relapse prevention during the latent phase of hypnozoites in the liver [2, 71, 139, 156]. Hence, Miatmoko et al*.* [139] engineered liposomes loading combination of chloroquine and primaquine. The physicochemical characteristics of liposomes and the rate of release of these two drugs from the liposomal formulations were investigated. These liposomes were made of HSPC, DSPE-mPEG200 and cholesterol (see Table 1). All the formulations showed sized lower than 150 nm, which is of interest for targeting hepatocytes. The in vitro drug released from liposomes indicated that ~ 20% of chloroquine and 44% of primaquine were released over 48 h from the liposomes loaded with the two drugs. These values were lower than that of the single drug-loaded liposomes, which displayed 40% of chloroquine and 63% of primaquine, respectively [139].

#### Liposomes for the treatment of cerebral malaria

Administered at low doses, the monomeric recombinant human tumour necrosis factor (rhTNF) protects mice against experimental cerebral malaria. In contrast, high doses of rhTNF induce cerebral pathology in malaria-infected mice [32, 157]. To increase the protective efficacy of rhTNF against *P. berghei*-induced experimental cerebral malaria in mice, Postma et al*.* [158] covalently coupled rhTNF to the outer surface of preformed conventional and PEGylated liposomes. The efficacies of liposome-conjugated rhTNF were compared to those of liposome-encapsulating rhTNF (and free rhTNF). After intravenous injection in mice, rhTNF conjugated to liposomes exhibited a significant reduction of parasitaemia and achieved greater protection against experimental cerebral malaria in comparison with free rhTNF. However, encapsulation of rhTNF into liposomes did not improve its protective efficacy against *P. berghei*-induced experimental cerebral malaria, suggesting that the liposomal bilayer stabilizes the bioactive trimeric configuration of rhTNF [158].

Betamethasone hemisuccinate, a water-soluble glucocorticosteroid with unfavourable pharmacokinetics, has potential roles in the experimental cerebral malaria [159–161]. Since passive targeting via nanosterically stabilized liposomes (nSSL) may increase permeability of vasculature in inflamed tissues and thus improve drug delivery to injured areas of the brain [162], Barenholz, Golenser and colleagues encapsulated betamethasone hemisuccinate using HSPC, cholesterol and DSPE-mPEG2000 at a mole ratio of 55:40:5 [132, 133]. To obtain a high and stable drug remote loading efficiency, a high drug-to-lipid ratio, and a unique slow controlled drug release (zero order), betamethasone hemisuccinate was loaded into these PEGylated nanoliposomes by a transmembrane calcium acetate gradient [132].

In experimental cerebral malaria in mice, injectable treatment with free betamethasone hemisuccinate at a dose of 5–20 mg/kg resulted in significant acute toxicity while this toxicity was completely abolished after encapsulation of the drug in nSSL [132]. Moreover, the intra-peritoneal administration of betamethasone hemisuccinate in nSSL effectively delivered and released the drug in brain thereby delaying the appearance of clinical signs and improving drug efficacy. Indeed, at 10 mg/kg on day 3, 5, 7 and 9 post-infection, betamethasone hemisuccinate nanoliposomes reduced the average clinical score to 2.7 ± 0.8 versus 7.5 ± 1.6 in the free betamethasone hemisuccinate-treated group and 14.5 ± 1.5 in the non-treated group. In addition to this, the incidence of cerebral malaria after treatment with 5 mg/kg/day of betamethasone hemisuccinate loaded in PEGylated nanoliposomes was similar to that seen upon administration of 20 mg/kg/day of free drug [132]. While artemisone monotherapy (2 × 20 mg/kg, day 11–15 post-infection, intra-peritoneal) was ineffective in preventing experimental cerebral malaria in mice, the combination of artemisone with nSSL-betamethasone hemisuccinate nano-drug was found to be highly efficacious for the treatment of experimental cerebral malaria, making it attractive for further investigation [132, 133].

Ghosh and colleagues demonstrated that maduramicin-loaded PEGylated liposomes have prophylactic (4 mg/kg, single dose) and therapeutic (1.5 mg/kg × 4 days) efficacies in *P. berghei*-infected mice [138]. To fulfill their data, the authors also evaluated the potential of these liposomes to clear the experimental cerebral malaria in mice. Interestingly, after administration to mice infected with *P. berghei*, maduramicin-loaded liposomes (1.5 mg/kg, day 2–5, subcutaneous) led to 100% chemosuppression (0% parasitaemia) on day 12 post-infection and a median survival time of > 45 days. While 37% parasitaemia was observed in the untreated group of mice (median survival time = 13 days), there was a parasite load of 15% in mice treated with free maduramicin (median survival time = 29 days). In comparison with the untreated mice, the long circulating liposomal maduramicin also suppressed (or maintained at basal levels) the key inflammatory markers (e.g. granzyme B), pro-inflammatory cytokines (e.g. TNF and interferon gamma) and adhesion molecules (e.g. intercellular adhesion molecule (ICAM-1) associated with the progression of experimental cerebral malaria. Moreover, maduramicin in PEGylated liposomal formulation increased intracellular reactive oxygen species (ROS) production and subsequent perturbation of parasite mitochondrial membrane potential [138].

### Liposome as active targeting drug delivery in malaria therapy

#### Liposomes for the treatment of intra-hepatic stage of malaria

To treat sporozoite-induced murine malaria, Alving et al*.* [163] engineered liposomes containing ceramide alone or neutral glycolipids such as cerebrosides (galactosylceramide, glucosylceramide, sulfogalactosylceramide), gangliosides (GM1, lactosylceramide) and phosphocholine ceramide (sphingomyelin) (see Table 2 for the different compositions). Interestingly, drug-free liposomes containing galactosyl-, glucosyl- or lactosylceramide interfere with the malarial life cycle during the liver stage and prevent the appearance of erythrocytic forms of malaria in mice infected with sporozoites [163]. In fact, up to 85–95% of sporozoites-infected mice treated with these liposomes were cured, while the cure rate recorded for the untreated mice was about 20%. On the other hand, liposomes containing sphingomyelin, sulfogalactosylceramide, ganglioside GM1 or ceramide alone exhibited no anti-malarial efficacy in mice. Moreover, all the glycolipid-liposomes had no effect on intra-erythrocytic stages of malaria in mice. These phenomenon may be explained by the fact that certain membrane glycolipids are associated with mammalian cells via glycolipid-lectin interactions and fusion thereby likely resulting in competition of liposomes (lipid phase) with sporozoites for the same receptor [163]. When primaquine was encapsulated in these galactosyl glycolipid-bearing liposomes for targeting hepatocytes via a galactose-binding lectin, its efficacy was enhanced by more than 46,000 times, thereby achieving a curative effect in mice at reduced doses [164]**.**

**Table 2:** Composition of liposomes used as active targeting drug delivery systems in malaria therapy

Drugs	Lipid composition	Ratio^a^	Ligands	References
–	DMPC: cholesterol: dicetylphosphate PC: sphingomyelin: cholestrol: dicetylphosphate	1:0.7:0.11 0.8:0.2:0.75:0.11	Glycolipids	[163]
–	EPC: cholesterol: gangliosides	20:20:4	Anti-rat erythrocyte F(ab’)_2_	[165]
–	EPC: cholesterol	1:1	Tuftsin derivatives	[166]
Chloroquine	EPC: cholesterol: gangliosides	20:20:4	Anti-mouse erythrocyte F(ab’)_2_	[167]
Chloroquine	PC: PS: cholesterol PC: PS: cholesterol: MPB-PE	9.5:1:10 9.5:1:10:0.5	Anti-mouse erythrocyte F(ab’)_2_	[168–170]
Chloroquine	EPC: cholesterol: gangliosides	20:20:4	Anti-mouse erythrocyte F(ab’)_2_	[171]
Chloroquine	EPC: cholesterol: gangliosides	20:20:4	MAbF_10_ MAbD_2_	[140]
–	Lipid di22: 1-PC(1,2-dieucoyl-sn-3-PC), Lipid-PEG-conjugate di22: 1-PE-PEG5000(1,2-dierucoyl-sn-3-(PEG5000)-PE, Lipid-PEG-peptide-conjugate di22: 1-AP-PEG3400-peptide(1,2-dierucoyl-sn-3-(PEG3400-succinyl-peptide)-aminopropane, Fluorescent lipid di22: 1-AP-Bodipy-TR-X(1,2-dierucoyl-sn-3-(Bodipy-TR-X)-aminopropane)	82:10:4:4^b^ 87:7:2:4 91:4:1:4 86:10:4:0	19-Aminopeptide from the CSP of *P. berghei*	[172–174]
Chloroquine Fluorescent probe pyranine 655 ITK® carboxyl quantum dots	DOPC: cholesterol DOPC: cholesterol: MPB-PE	80:20 77.5:20:2.5 80:20	BM1234	[175]
Chloroquine Fosmidomycin	DOPC: cholesterol DOPC: cholesterol: MPB-PE	80:20 77.5:20:2.5	BM1234	[176]
Chloroquine	DOPC: cholesterol DSPC: cholesterol DOPC: cholesterol: DSPE-PEG-Mal DSPC: cholesterol: DSPE-PEG-Mal DOPC: cholesterol: MPB-PE	80:20 90:10 75:20:5 85:10:5 65:20:15	Anti-MAHRP1_21-40_ Anti-HRP_2_ Anti-GPA	[177]
–	DSP: cholesterol: DSPE-mPEG	2:1:0.1	PSP	[24]
Lumefantrine	DSPC: cholesterol: DSPE-mPEG2000	85:10:5	NTS-DBL1α N-terminal domain of a rosetting PfEMP1	[178]
Chloroquine, 7c, 7d (4-aminoquinoline compounds) Primaquine Quinine, BCN-01, BCN-02 (aminoalcohol compounds) Tafenoquine	DSPC: cholesterol: DSPE-PEG2000-Mal DOPC: cholesterol: DSPE-PEG2000-Mal	85:10:5	Mouse monoclonal IgG2b anti-human GPA (SM3141P) Rat monoclonal IgG2b anti-mouse TER-119 (AM31858PU-N)	[179]
Primaquine	DOPC: PE: cholesterol: DOTAP	46:30:20:4	Heparin	[180]
Pyronaridine + Atovaquone	DOPC: cholesterol: DSPE-PEG2000 POPC: cholesterol: DSPE-PEG2000 DSPC: cholesterol: DSPE-PEG2000	75:20:5	Anti-GPA	[181]
Poupartone B	DOPC: cholesterol: DOTAP	76:20:4	Heparin	[182]

^a^Unless otherwise indicated, the ratio is expressed in molar

^b^The value corresponds to the molar ratio of lipid, lipid-PEG-conjugate, lipid-PEG-peptide conjugate and fluorescent lipid, respectively

Taking into account the above-mentioned results, Gupta’s research group designed liposomes decorated with tuftsin and its derivative for the treatment of malaria liver stage [107, 166]. The hydrophilic tuftsin, a natural tetrapeptide (threonine-lysine-proline-arginine) and its hydrophobic derivative (Thr-Lys-Pro-Arg-NH-(CH_2_)_2_-NH-CO-C_15_H_31_) are macrophage activators [107, 166]. As previously reported, activated macrophages showed enhanced killing activity on intraerythrocytic malarial parasites [25, 107, 144, 166, 183]. Interestingly, pre-treatment of *P. berghei*-infected mice with the hydrophobic derivative of tuftsin (50–100 µg/animal, intravenous administration on days − 3 to 0) conjugated to EPC-cholesterol liposomes (1:1, molar ratio) exhibited higher prophylactic anti-malarial efficacy than that of free tuftsin derivative [166]. Indeed, parasitaemia and mortality rate in the group of mice pre-treated with liposomal tuftsin decreased significantly, as compared to those pre-treated with either unloaded-liposomes or free tuftsin derivative. Surprisingly, curative treatment with these tuftsin-bearing liposomes (50–100 µg of ligand/animal on days 0 to + 3) did not confer much efficacy [166].

Longmuir, Robertson and colleagues engineered dierucoyl phosphatidylcholine (DEPC)-based liposomes decorated with the 19-amino-acid sequence of the circumsporozoite protein (CSP) [172]. The composition of these liposomes is shown in Table 2. They consisted of lipid, lipid-PEG-conjugate, lipid-PEG-peptide conjugate and fluorescent lipid (82:10:4:4, molar ratio). It should be remembered that the intra-hepatic malarial infections and the malaria recrudescence caused by sporozoites are, inter alia, attributed to the 19-amino-acid sequence of the circumsporozoite protein (CSP) exhibiting a liver-targeting specificity. Located at the surface of liver-stage parasite, this protein can bind to hepatocytes via the heparan sulfate proteoglycans (HSPGs) [184, 185]. When administered intravenously into mice, the developed peptide-containing liposomes were rapidly cleared from the circulation and were recovered almost entirely in the liver (>80%) [172]. The accumulation of these liposomes in the liver was several 100-fold higher compared to heart, lung and kidney, and more than tenfold higher compared to spleen [174].

In another study, Longmuir, Robertson and co-workers evaluated the influence of composition of 19-aminopeptide-containing liposomes in their specificity to target liver. So, the authors systematically varied the mole fractions of lipid (82–91 mol%), lipid-PEG-conjugate and lipid-PEG-peptide conjugate (see Table 2) [173]. The selected formulations exhibited effective liposome targeting to the liver, with approximately 80% of the total injected dose recovered in the liver within 15 min, in agreement with previous results [174]. Moreover, uptake of these liposomes by liver cells was more than 600-fold higher than uptake by those in the heart, and more than 200-fold higher than uptake by lung or kidney cells. Additionally, this targeting to liver was effective upon repeated (up to three) administrations to the host at 14-day intervals [173].

#### Liposomes for the treatment of blood stage of malaria

In the 1980s, Gupta and his group initiated, for the first time, a vast project based on the development of antibody-mediated targeting of liposomes to red cells. In one of their first studies, they prepared anti-rat erythrocyte F(ab’)_2_ bearing liposomes from EPC, cholesterol and gangliosides (see Table 2) [165]. After intravenous injection in rats, the covalent attachment of F(ab’)_2_ enhanced specifically the binding of liposomes to erythrocytes. Indeed, about 20% of these target-specific liposomes were associated with the erythrocytes [165]. This binding was extended up to 3 h, reducing the blood clearance rate of the liposomes without affecting the survival time of the erythrocytes. Additionally, an appreciable decrease in the uptake of these F(ab’)_2_-bearing liposomes by no target tissues (for example liver, spleen and plasma) was observed [165].

As a result of these promising outcomes, Gupta and co-workers evaluated whether anti-mouse erythrocyte F(ab’)_2_ bearing liposomes could be effective as vehicles for delivering chloroquine to erythrocytes in vivo [167]. After administration to *P. berghei*-infected mice, 15–20% of the injected dose of liposomes coupled to cell-specified antibody bound to the erythrocytes [167], which agreed with previous results [165]. Moreover, 20–30% of these cell-targeting liposomes deliver their contents to the target cells [167]. Despite their poor antibody recognition of target cells, a single 5 mg/kg dose of chloroquine loaded anti-erythrocytes F(ab’)_2_-bearing liposomes was found to be more effective than F(ab’)_2_-free liposomes and free chloroquine in suppressing both chloroquine-sensitive and chloroquine-resistant *P. berghei* infections in mice [167, 171]. Additionally, this targeted liposomal formulation increased the therapeutic efficacy of chloroquine and prolonged the survival time of the treated animals at least up to day 12 post-chloroquine-resistant infection [167, 171]. This confirmed the ability of these antibody-targeted liposomes to partly concentrate the drug in erythrocytes. Of note, Crommelin and colleagues have previously shown that encapsulation of chloroquine in non-targeted liposomes could increase the effectiveness of this drug against chloroquine-resistant *P. berghei* infections, but this required a daily dose of 400 mg/kg (i.e. 8 mg/mouse of 20 g) for 3 consecutive days to achieve 90% survival with no recurrent infection [117]; this corresponds to 80 times greater dose than the dose of chloroquine encapsulated in the immunoliposomes developed by Gupta and colleagues [167, 171].

To further increase the cell-specifity of immunoliposomes, their fate in target cells (RBCs) and their pharmacological activity, Crommelin et al*.* engineered drug-free immunoliposomes of the F(ab’)_2_-MPB-PE-REV type that were made by covalently linking F(ab’)_2_ fragments (from rabbit antimouse erythrocyte IgG) to reverse-phase evaporation vesicles (REV) via maleimido-4-(*p*-phenylbutyrate)phosphatidylethanolamine) (MPB-PE) as anchor molecule (Table 2) [168, 169]. Data revealed that, at equal protein doses (~ 130 µg), the unloaded F(ab’)_2_-liposomes intravenously injected in rat induced a faster elimination of the RBCs from the bloodstream and higher uptake into the spleen than the free F(ab’)_2_. Nevertheless, the fact that the doses of F(ab’)_2_-liposomes and free F(ab’)_2_ taken up into the liver (hepatocytes) was lower than those into the spleen constitute an important limitation suggesting an improvement of their specificity [169]. In addition to this, chloroquine-loaded F(ab’)_2_-MPB-PE-REV liposomes (0.8 mg of drug per rat) were significantly more effective than the non-targeted liposomes encapsulated chloroquine, free-MPB-PE-REV liposomes and free chloroquine in preventing or delaying a patent infection of non-synchronized pRBC in rats [169, 170]. Indeed, parasitaemia became patent about 9.6 days, 6.4 days, 4.4 days and 6.2 days after infection in mice treated with the chloroquine immunoliposomes, chloroquine liposomes, free immunoliposomes and free chloroquine, respectively [170]. By contrast, when the treatment was given to rats infected with synchronized pRBC (reticulocytes), the therapeutic efficacy of these chloroquine immunoliposomes was significantly improved [170].

In another study, Crommelin et al*.* evaluated the therapeutic efficacy of chloroquine loaded in immunoliposomes with rabbit anti-mouse RBCs (anti-mRBC) F(ab’)_2_-liposomes in rats infected with early stage of *P. berghei* infected cells (> 90% trophozoites). Due to the absence of mature schizonts in these synchronized parasitized reticulocytes, the latter are not able to release free parasites in the bloodstream. Interestingly, intravenous administration of chloroquine-loaded anti-mRBC F(ab’)_2_-liposomes led to greater survival rate of the infected rats in comparison with free chloroquine: 33% versus 0%, respectively, while both used at a dose of chloroquine equivalent to ca. 12 mg/kg [119].

Considering all the data presented above, researchers hypothesized that the therapeutic efficacy of anti-malarial drugs could be more pronounced if (i) the performance of targeted liposomes in specific pRBC recognition was better, (ii) the developed immunoliposomes could also bind to merozoites and free parasites (iii) the capacity of liposomes to encapsulate drugs as well as their endocytic activity at the surface of RBCs and pRBCs were increased [167, 170].

Hence, to increase the survival rate and the survival time of the treated mice, Gupta and co-investigators looked for more specific ligands that could better deliver the drug in the biophase in comparison with anti-mouse erythrocyte F(ab’)_2_ [140]. For this purpose, they covalently attached F(ab’)_2_ fragments of the monoclonal antibodies MAbF_10_ and MAbD_2_ to the surfaces of liposomes (see composition in Table 2). After assessing their binding specificities, it was found that MAbF_10_-liposomes interacted in vitro with normal (< 3%) and *P. berghei*-infected (~ 16%) mouse erythrocytes [140]. By contrast, the maximum binding of MAbD_2_-liposomes was 17–20% with both normal and *P. berghei*-infected erythrocytes. These findings suggested that grafting of MAbF_10_ on the liposome surface was more specific to *P. berghei*-infected erythrocytes than that of MAbD_2_-liposomes [140]. After intravenous administration in mice infected with a chloroquine-resistant strain, MAbF_10_ liposomes containing chloroquine exhibited considerably higher levels of anti-malarial activity than chloroquine-MAbD_2_-liposomes. Indeed, at a dosage of 5 mg/kg per day on days 4 and 6 post-infection, MAbF_10_-liposomes and MAbD_2_-liposomes containing chloroquine cured, respectively, 75–90% and 40–50% of the animals on day 30 post-infection [140]. These findings confirmed that the therapeutic efficacy of chloroquine was markedly increased through targeted liposomal drug delivery.

Fernàndez-Busquets and co-workers also engineered immunoliposomal nanovectors that were able to target more specifically pRBCs and release their contents inside these cells, but not in non-parasitized RBCs [175]. For this purpose, the authors prepared liposomes using the mixture of 1,2-dioleoyl-*sn*-glycero-3-phosphocholine (DOPC): cholesterol: MPB-PE (77.5:20:2.5). The resulting liposomes were covalently functionalized with about 5 molecules of BM1234, an oriented, specific targeting monoclonal antibody (which is also known as the membrane-associated histidine rich protein 1, MAHRP1) [177]. This antigen is specific for pRBCs infected by the late forms of *Plasmodium* species (trophozoites and schizonts), but not for ring-stage pRBCs. In living *P. falciparum* cultures, these developed immunoliposomal nanovectors recognized 100% of late form-containing pRBCs (and 0% of non-infected RBCs) and infiltrated their content in the host cells in less than 90 min [175]. Consequently, 2 nM chloroquine delivered inside targeted immunoliposomes cleared ~ 27% of pRBCs (versus 50% of pRBCs for free chloroquine at 20 nM) thereby suggesting an improvement in terms of drug efficacy. Liposomes not functionalized with antibodies were also specifically directed to pRBCs, although with less affinity than immunoliposomes [175].

In further studies, Fernàndez-Busquets and collaborators quantified the efficiency of these nanovectors bearing 5 BM1234 units in ameliorating the anti-malarial activity of both chloroquine and fosmidomycin (see composition in Table 2) [176]. Liposomes containing either chloroquine (1.6 nM) or fosmidomycin (325 nM) reduced in vitro parasitaemia by 10% when added at the ring stage and by 26.5% when added at the trophozoite stage, which was in agreement with previous results [175]. In contrast, free chloroquine (2 nM) or fosmidomycin (360 nM) reduced parasitaemia by 3–6% when added at either the ring or the trophozoite stage. On the other hand, 20 nM of free chloroquine were necessary for killing 28% of trophozoites [176]. Consequently, liposomes covalently functionalized with an average of 5 half-antibodies BM1234 improved by tenfold the therapeutic effects of these two anti-malarial drugs [175, 176]. In addition to this, immunoliposomes bearing 5, 50 or 250 BM1234 units and encapsulating 4 nM chloroquine led to a reduction of 30, 43 and 51% parasitaemia (trophozoite stage), respectively [176]. All these results confirmed that the antibody-functionalized liposomal nanovectors for the targeted delivery of drugs specifically to pRBCs have shown complete discrimination in vitro for pRBCs vs. non-infected RBCs [175, 176]. Of note, the best performing immunoliposomes were those added to *Plasmodium* cultures having a larger number of late form-containing pRBCs (i.e. trophozoites and schizonts), consistent with the previous studies [175]. These findings revealed also that increasing the number of antibodies on the surface of immunoliposomes may improve their in vivo performance [175, 176].

Notably, the above-mentioned data on antibody-targeted liposomes for malaria therapy show tremendous progress in this field. However, the anti-malarial effect obtained was mainly attributed to the encapsulation of anti-malarial drugs in liposomes and partially to antibody targeting. To reverse this balance, Fernàndez-Busquets and his team tested the capacity of anti-glycophorin A (anti-GPA), anti-histidine-rich protein 2 (anti-HRP2) and anti-MAHRP1_21-40_ as targeting agents for the functionalization of immunoliposomes (see composition in Table 2) [177]. Anti-GPA and anti-HRP2 were respectively raised against GPA and HRP2 that are located on erythrocyte surfaces while anti-MAHRP1_21-40_ was targeted against the intracellular MAHRP1 antigen. All the three selected antibodies were compared to BM1234 in terms of anti-malarial efficacy [177]. To facilitate the coupling reaction of antigen to targeting antibodies and improve the targeting properties of the anti-GLA liposome formulations, a lipid bearing PEG 2000 linker terminated with a maleimide group (Mal) was included in their lipid bilayer. Depending on the chemical group (thiol, carbohydrate or primary amine) of the half-antibodies that binds to the maleimide group, three types of immunoliposomes were designed, namely immunoliposome-PEG-Mal-SH-Ab, immunoliposome-PEG-Mal-CHO-Ab and immunoliposome-PEG-Mal-NH_2_-Ab. The results from in vitro studies indicated that free anti-GPA were able to recognize the entire RBC and pRBC populations whereas the 3 others antibodies bound less than 1% of pRBCs [177]. Consistently with these results, anti-GLA immunoliposomes were able of targeting 100% RBCs and pRBCs after only 15 min exposure time to *P. falciparum* cultures. The efficiency of cell targeting of these formulations was the highest with immunoliposome-PEG-Mal-NH_2_-Ab and the lowest with immunoliposome-PEG-Mal-SH-Ab. Interestingly, when encapsulated with 50 nM of chloroquine, these immunoliposomes completely inhibited the in vitro growth of early stages of *P. falciparum* 3D7 strain. In contrast, 200 nM of free chloroquine had no significant effect on the viability of this parasite [177]. In addition to this, the immunoliposomes bearing antibodies against the RBC surface protein glycophorin A permitted to transfer efficiently the anti-malarial drug into non-parasitized RBCs. By doing so, the parasite that entered the host cells instantly encountered the drug, which compromised its growth and survival capacity. Intracellular delivery of the encapsulated cargo may mainly occur following a sustained release process that take place at the targeted cell surface [177]. Finally, during preliminary in vivo studies, data demonstrated that, when administered intravenously at the dose of 0.5 mg/kg for 4 days, chloroquine loaded in immunoliposome-PEG-Mal-NH_2_-Ab completely cleared *P. falciparum* in mice grafted with human erythrocytes. This efficacy was 40- and 100-fold higher than that of free chloroquine (1.75 mg/kg × 4 days) and chloroquine in non-target liposomes (0.5 mg/kg × 4 days), respectively. Based on these data, the authors suggested that anti-GLA-immunoliposomes actively loaded with chloroquine may act as prophylactic and therapeutic agents by simultaneously delivering the drug to both infected and non-infected RBCs [177].

Based on this approach, hydrophilic (pyronaridine) and lipophilic (atovaquone) anti-malarial drugs were encapsulated, alone or in combination, in GPA-targeted immuno-PEG-liposome [181]. The developed immunoliposomes that consisted of 1-palmitoyl-2-oleoyl-glycero-3-phosphocholine (POPC), cholesterol and DSPE-mPEG2000-Mal were obtained by coupling anti-GPA antibody to the maleimide group as mentioned above (Table 2) [177]. While at concentration of 137.2 nM, the immunoliposomized pyronaridine resulted in 50% inhibition of the in vitro growth of the parasite, pyronaridine-loaded liposomes and free pyronaridine exhibited significantly lower antiplasmodial activity (16.2% and 2% inhibition, respectively). The same trend was observed for atovaquone which showed an IC_50_ of 43.1 nM after incorporation in immunoliposomes [181]. Nonetheless, the co-encapsulation of these two drugs in the immunoPEGliposomes required higher amount of drugs (379 nM for pyronaridine and 116 nm for atovaquone) for inhibiting 50% of growth of malaria parasite, suggesting that this particular drug combination is antagonistic and less active than when these individual drugs are used separately [181].

Noteworthy, the outstanding performance of chloroquine-anti-GLA-liposomes was achieved in humanized and immunosuppressed mice infected with uncomplicated falciparum malaria (< 3% parasitaemia) [177]. To overcome this feebleness, Fernàndez-Busquets and co-workers evaluated the ability of anti-GLA-targeted liposomes containing different aminoalcohol- and aminoquinoline-type drugs to display simultaneous prophylactic and therapeutic activities in fully immunocompetent mice infected with a lethal strain of *Plasmodium yoelii* [179]. Indeed, in comparison with the humanized mice, this murine malaria model offered a cost-effective alternative for the in vivo study of immunoliposome-based severe malaria therapies. The lipid phase of the developed liposomes contained DOPC, an unsaturated type of phosphatidylcholine or DSPC, which is a saturated-type of phosphatidylcholine that reduces membrane fluidity and permeability (see composition in Table 2). Based on their physicochemical properties, DOPC and DSPC allowed the encapsulation of lipophilic agents and hydrophilic drug, respectively. On the other hand, since considerable cell-agglutinating events were observed with immunoliposomes bearing mouse monoclonal IgG2b anti-human GPA (SM3141P) (> 10 µM lipid amounts), the authors also evaluated the potential of rat monoclonal IgG2b anti-mouse TER-119 (AM31858PU-N) antibodies to be used as ligand agents for generating immunoliposomes [179]. The TER 119 marker was specifically expressed from the early proerythroblast to mature mouse RBC stages [186, 187]. Data from in vitro studies indicated that, upon encapsulation into the GPA-immunoliposome targeting all RBCs, the compound 7c, a chloroquine analogue, was efficiently delivered to RBCs infected with chloroquine-sensitive and chloroquine-resistant *P. falciparum* strains after only 15 min of exposure [179]. With a high in vitro targeting efficacies to both naïve and *Plasmodium*-infected-RBCs and retention yields of ~ 100% in agreement with previous reports [177], these 7c-immunoliposomes exhibited IC_50_ values of 48.6 nM (late forms of resistant parasites) and 100.7 nM (late forms of sensitive parasites), that were 12 to 27-fold lower than that of the free drug.

When intravenously administered to fully immunocompetent mice infected with *P. yoelii*, 7c-anti-GPA-liposomes (20 mg/kg over 4 days) exhibited a significant parasitaemia reduction (45–55%). On the other hand, two minutes after its intravenous administration (5 mg/kg on day 1, 2.5 mg/kg on day 2 and 1 mg/kg on day 3), the 7c drug encapsulated in TER119-immunoliposomes targeted both RBCs and pRBCs, and reached retention efficacies of > 95% thereby decreasing blood parasitaemia from severe to uncomplicated malaria (i.e. from > 25% to < 5%) in *P. yoelii*-infected immunocompetent mice [179].

To prevent microvascular sequestration of pRBC, which is strongly linked to their cytoadherence (i.e. adherence on vasculature endothelium) and rosetting (i.e. adherence on non-parasitized RBCs), Fernàndez-Busquets et al*.* developed lumefantrine-loaded immunoliposomes functionalized with a polyclonal anti-rosetting antibody against the NTS-DBL1α N-terminal domain of a rosetting *P. falciparum* erythrocyte membrane (PfEMP1) variant. The composition of immunoliposomes is shown in Table 2. After 30 min of incubation in *P. falciparum* cultures, the immunoliposomes containing 2 µM of lumefantrine showed a 60% growth inhibition for rosettes (i.e. pRBCs with a rosetting phenotype) and a reduction in ca. 70% of pRBCs containing all the parasitic forms [178]. This approach is of interest since rosettes facilitate the occlusion of microvascular blood vessels, which obstructs blood flow leading to hypoxia, metabolic acidosis and other phenomena involved in the pathophysiology of malaria [21, 188, 189].

Because anionic lipids such as phosphatidylserine (PS) move from the inner membrane of erythrocytes to their outer surfaces during eryptosis, Tagami et al*.* [24] engineered liposomes conjugated to a PS-specific peptide (PSP). The developed PSP-liposomes were composed of DSPC: cholesterol and PSP-DSPE-mPEG2000 (2:1:0.1, molar ratio). Interestingly, the binding of PSP-liposomes to eryptosis-induced RBCs (eRBCs) was significantly higher than those of RBCs. The specific binding of PSP-liposomes to eRBCs occurred within 3 h post-incubation. These findings suggest that PSP-conjugated liposomes could be an effective targeted nanocarrier drug delivery system for treating eRBCs and different malaria-infected RBCs (unlike liposomes coupled with monoclonal targeting antibodies) [24].

Heparin acts as an anti-malarial drug that blocks merozoite invasion of erythrocytes [190], but do not influence the clinical course in human falciparum malaria [144, 191]. Even though no anti-malarial resistance to heparin has been described so far, it has been abandoned in therapeutic because of its strong anticoagulant action that was associated with intracranial bleeding [190]. Nevertheless, heparin and others glycosaminoglycans like heparan sulfate and chondroitin sulfate have specific binding affinity for pRBC (vs. non-infected RBCs), which make them interesting alternatives to antibody-mediated targeting [190, 192–194]. This is why Fernàndez-Busquets and colleagues engineered liposomes bearing heparin or its related polysaccharides (see composition in Table 2) [180]. Although the heparin-related compounds are recognized as pRBC-binding molecules, their attachment on the liposome surface requires careful optimization to achieve good reproducibility and stability [180, 194–196]. In contrary, heparin that targets mainly trophozoite- and schizont-infected RBCs stages was successfully adsorbed onto positively charged liposomes via electrostatic interactions [180, 197, 198]. Used at non-anticoagulating concentrations, these heparin-bearing liposomes enhanced three-fold the antiplasmodial activity of encapsulated primaquine in in vitro* P. falciparum* cultures, and provided potent parasiticidal activity in mice [180]. Apart from these significant targeting and pharmacological activities, the heparin-based approach is very interesting in economical point of view since heparin is about 10 times more affordable than monoclonal antibodies [180].

Recently, poupartone B, a promising anti-malarial compound isolated from *Poupartia borbonica*, was encapsulated in liposomes coated with heparin and tested on artemisinin-resistant *P. falciparum* isolate [182]. The composition of this liposomal formulation is shown in Table 2. The poupartone B-engineered liposomes exhibited IC_50_ value of 0.41 µg/ml (versus 0.69 µg/ml for the free drug). Moreover, the liposomal formulation improved the selectivity index 2 times in vitro and proved to be 3 times less toxic than the unloaded poupartone B in a zebrafish (*Danio rerio*) model [182].

### Liposomes as tool for diagnosis and therapeutic monitoring of malaria

Portnoy et al*.* [136] used liposomes containing the FDA-approved fluorescent dye indocyanine green (ICG) in combination with artemisone solution to address the urgent need for a simple, non-invasive imaging methodology for early diagnosis and therapeutic monitoring of cerebral malaria. The liposomal formulations were composed of Phospholipon S75 (SPC) and cholesterol (with or without DSPE-mPEG2000) (Table 1). After induction of cerebral malaria, the infected mice were treated with artemisone solution (5 mg/kg, injected intraperitoneally, on days 3–5 post-infection). Thereafter, on day 6 post-infection, the mice were injected with ICG-liposomes (200 µl, 8 mg/kg, intraperitoneal) and then repetitively scanned using an in vivo imaging system. Interestingly, liposomal ICG demonstrated increased emission intensity in comparison with free ICG. The emission of ICG liposomes in brain of untreated mice was greater than that of artemisone-treated mice and naïve mice. According to the histological data, the accumulation of ICG-liposomes in the cerebral vasculature may be due to extensive uptake mediated by activated phagocytes [136]. Overall, the ICG liposomes may offer a valuable diagnostic tool, serving as a biomarker for treatment follow up in cerebral malaria.

### Liposomes as adjuvant and carrier for malaria vaccines

Liposomes were first proposed as immunological adjuvants by Gregoriadis and Allison in 1974 [199, 200], after being used to study the physical behaviour of biological membranes [100, 201, 202] and then as drug carriers [112]. Indeed, extensive research has shown that liposomes have a high potential as carriers of antigens, synthetic peptides, and immunostimulants [203–206]. In fact, when paired with antigens, liposome-based adjuvants improve the vaccine’s immunogenicity, resulting in long-term protection [204]. However, it should be noted that liposome design guidelines must be tailored in order to achieve or improve the adjuvant properties and desired immune responses of these adjuvant systems. This includes, for example, the use of appropriate lipid composition, optimal surface charge, antigen loading capacity, stealth behaviour, and the proper selection of immunomodulators and route of administration [207–211]**.**

Several studies have found that liposomes are an appealing platform for malaria vaccine development due to their safety profile, biocompatibility, biodegradability, versatility, and plasticity [207–211]. As part of a health strategy for protection of its personnel, the Malaria Vaccine Branch of the U.S. Walter Reed Army Institute of Research (WRAIR) has played an historical and important role in the development of liposome-based adjuvants and liposome-containing vaccines**.** Indeed, the U.S. Army was ranked second among organizations involved in vaccine research and development [212–215].

A worthy mention is the WRAIR designed, manufactured and tested numerous vaccine adjuvants which include the Army Liposome Formulation (ALF) as well as the ALFA, ALFQ and ALFQA adjuvanted liposomes that provide innovative, potent, and safe innate immunity for vaccines. The ALF adjuvant contains saturated phospholipids (20.61 mM DMPC and 2.29 mM DMPG), 14 mM cholesterol, and 0.13 mM monophosphoryl lipid A (MPLA) [204, 216–219]. Lipid A is the lipid component of lipopolysaccharides (or endotoxins), large molecules found in the outer membrane of gram-negative bacteria. Lipid A is responsible for the endotoxic activity and immunostimulant activity of lipopolysaccharides (LPS). MPLA is a low-toxic derivative of the lipid A region of lipopolysaccharides that boosts adaptive immunity [220].

ALFA is referred to ALF-type liposomes containing adsorbed aluminum hydroxide (alum) gel while ALFQ is the combination of ALF-based liposomes with high amounts of cholesterol and the immunostimulant QS-21, a triterpenoid glycoside saponin derived from the bark of the *Quillaja saponaria*, fraction 21 (see Table 3). ALFQA is refereed to ALF plus QS-21 and alum [204, 221]. Interestingly, the ALF-based vaccine adjuvants have shown promise for enabling creation of vaccine candidates against *P. falciparum* malaria [204, 222]. Indeed, the ALF-type liposomes containing MPLA exhibited considerable adjuvant activity with a variety of antigens whereas the same liposomes lacking MPLA had little or no detectable adjuvant activity by themselves [220, 223, 224]. In addition to this, AS01 is another liposome-based adjuvant system (DMPC, DMPG and cholesterol) developed by GlaxoSmithKline Biologicals (GSK). It also contains 3′-*O*-desacyl-4′-monophosphoryl lipid A, a synthetic analog of MPLA and the QS-21 (see Table 3) [221, 225].

**Table 3:** Composition of liposomes-based vaccines for malaria prophylaxis

Antigens	Lipid composition	Ratio^a^	References
R32tet_32_	DMPC: cholesterol: dicetylphosphate: lipid A DMPC: cholesterol: DMPG: lipid A DMPC: cholesterol: dicetylphosphate: lipid A (± alum) DMPC: cholesterol: DMPG: lipid A (± alum)	1:0.75:0.1:0.02 0.9:0.75:0.1:0.02 1:0.75:0.1:0.02 (± 20 nmol/µmol of phospholipid) 0.9:0.75:0.1:0.02 (± 20 nmol/µmol of phospholipid)	[226, 227]
R32NS1_81_	Cholesterol: DMPC: DMPG (± MPLA)	0.75:0.9:0.1 (± 52.6 µg/µmol lipid)	[228]
R32NS1	DMPC: DMPG: cholesterol: lipid A	1.8:0.2:1.5:0.04 1.8:0.2:1.5:0.005	[229]
RTS,S	DMPC: cholesterol: DMPG (± MPLA)	0.9:0.75:0.1 (± 0.2 µg/dose)	[230]
66PyIMP	EPC: cholesterol	2:1	[231]
RTS,S	DOPC: cholesterol: MPLA: QS21^b^	1:250:50:50 (w/w)	[204, 223]
PfMSP-1_19_	SPC: Span 80	86:14 (w/w)	[232]
FMP013 FMP014	DMPC: DMPG: cholesterol: MPLA: QS21^c^	6986.27:788.73:5.413:200:100 (w/w)	[233, 234]
Pf25	DPPC: cholesterol: CoPoP: PHAD	4:2:1:1 (w/w)	[235]

^a^Unless otherwise indicated, the ratio is expressed in molar

^b^This mixture represent the composition of AS01-type liposome

^c^This mixture represent the composition of ALFQ-type liposome

#### Liposome-based vaccines for sporozoite-stage malaria

In the mid-1980s, the WRAIR had undertaken a major programme to develop an effective vaccine against sporozoite-induced malaria. In collaboration with the U.S. National Institutes of Health, they achieved the identification of the complete structure of the gene encoding the circumsporozoite protein (CSP) of *P. falciparum* [236]. Additionally, they identified, in the middle of the CSP, an immunodominant epitope containing repeating tetrapeptides that are capable of inducing protective immunity [236–238]. As part of this effort, both institutes developed two types of artificial antigen derived from the central repetitive region of the CSP of *P. falciparum* sporozoites, namely 16-mer peptide containing a repeating tetrapeptide (asparagine-alanine-asparagine-proline) and R32tet_32_, a genetically engineered protein from *Escherichia coli* that contain 32 tandem copies of a tetrapeptide sequence [237, 239].

To improve their cellular immunity responses, which are short-lived or suppressed during acute *P. falciparum* malaria [240, 241], 16-mer peptide and R32tet_32_ antigens were conjugated to carrier proteins (e.g. albumin) and then incorporated into liposomes [220, 226]. The liposomes used for immunization were multilamellar vesicles containing DMPC, cholesterol, dicetyl phosphate and lipid A (molar ratio of 1:0.75:0.1:0.02 at 0 weeks) or DMPG, cholesterol, dicetyl phosphate and lipid A (molar ratio of 0.9:0.75:0.1:0.02 at 4 weeks) [226]. After administration of these two liposomes (containing carrier protein conjugated peptide) in animals, the levels of antibodies to CSP were enhanced thus boosting their immune responses, but these were even higher with liposome-encapsulated R32tet_32_ [220, 226]. As mentioned above, these immunological effects were due to the incorporation of the adjuvant lipid A in the lipid bilayers of these liposomes [220, 226–228, 238, 242]. Indeed, the liposome R32tet_32_-lipid A-alum formulation showed even more enhanced efficacy than the R32tet_32_ adsorbed onto alum only (Falciparum Sporozoite-1 vaccine (FSV-1)^®^) [226, 241, 243].

R32NS1_81_ (also called R32NS1) is another recombinant antigen that contain epitopes of protective monoclonal antibodies to the central repeat region of the CSP of *P. falciparum*. Served as an efficient expression partner, NS1_81_ refers to a sequence of 81 amino acids of the influenza A virus nonstructural protein 1 [228]. Adsorbed with alum, this antigen had previously been found to be poorly immunogenic in humans [238]. Hence, to improve its humoral immune response, R32NS1_81_ was encapsulated in a variety of alum-adsorbed liposomal vaccine candidates that contained DMPC, DMPG, cholesterol (molar ratio, 0.9:0.1:0.75) and lipid A [or monophosphoryl lipid A (MPLA)]. The safety, immunogenicity and prophylactic activity of these injectable liposome-based vaccines was confirmed in human subjects [228].

In another experiment, Verma et al*.* [229] observed increased antigen expression by macrophages (macrophage activation) and an enhanced number of macrophages serving as antigen-presenting cells (antigen presentation) in mice after intra-peritoneal injection of R32NS1_81_ loaded in negatively-charged multilamellar liposomes. These liposomes were composed of DMPC, DMPG, cholesterol and lipid A in molar ratio of 1.8:0.2:1.5:0.005 (*Limulus* negative).

In the late 1980s, GlaxoSmithKline (GSK), in collaboration with WRAIR developed the RTS,S vaccines (RTS,S/AS01 and RTS,S/AS02) [204, 243]. The RTS,S/AS01 vaccine was engineered using the genes from the central repeat region (R) and the C-terminal region containing the T-cell epitopes of pre-eryhthrocytic CSP of the *P. falciparum* malaria parasite (T), and a viral envelope protein of the hepatitis B surface antigen (HBsAg) (S) to which was added the AS01 liposome adjuvant [222, 230, 244–247]. In contrary to the AS01-adjuvanted vaccine, AS02 is a squalene emulsion comprised of MPLA and QS-21 [222, 248]. Clinical trials of this recombinant protein-based malaria vaccine were performed over 4 years of follow up in sub-Saharan Africa [249, 250]. Depending on the age of the patients (infants, children or adults), the number of administered doses and the clinical malaria stage, the level of protection of RTS,S/AS01 vaccine ranged from 18 to 53%, which correspond to modest efficacy. This protection was very marked in the months following vaccination but, waned rapidly over the years until “negative efficacy” was reached after 5 years [248–257].

Recently, two synthetic candidate antigens namely Falciparum Malaria Protein-013 (FMP013), a nearly full-length recombinant CSP [258, 259] and FMP014, a self-assembling protein nanoparticle (SAPN) was developed by the WRAIR Malaria Vaccine Branch has produced [234]. To improve the immunogenicity of these candidate malaria vaccines, ALFQ was down-selected as an optimal adjuvant for phase 1 safety study [205] and controlled human malaria infection efficacy study in immunized volunteers [233].

#### Liposome-based vaccines for merozoites-stage malaria

As discussed above, merozoites invade and multiply within host erythrocytes during the life cycle of *Plasmodium* species in humans [15, 16, 25]. Therefore, the proteins that mediate the binding and evasion of erythrocytes by merozoites are also considered as attractive candidates for blood stage malaria vaccines. Among them, one can cite the *P. yoelii* merozoite surface protein-1 (PyMSP-1) and the *P. falciparum* merozoite surface protein-1 (PfMSP-1) that play an important role in RBC invasion. Indeed, antibodies naturally acquired and directed against these proteins or parasite ligands may block the invasion of erythrocytes, thus limiting the multiplication of the parasites and providing protection against clinical malaria [260, 261].

To improve the immune response of *P. yoelii* merozoite surface protein-1 (PyMSP1), it was encapsulated in liposomal formulations [262]. These liposomes were formulated with CAF01, a cationic adjuvant liposome formulation that comprises (i) dimethyldioctadecylammonium bromide (DDAB), a delivery vehicle and (ii) trehalose 6,6-dibehenate (TDB), a synthetic analog of mycobacterial cord factor (trehalose dimycolate) having immunomodulator properties [262]. In pre-clinical studies, immunization with CAF01-PyMSP1 resulted in significantly higher antibody and IFN-γ cytokine responses than immunization with the alum adjuvanted vaccine formulation. A significant protection against *P. yoelii* was also observed in CAF01-PyMSP1-immunized mice. Indeed, clearance of parasites from the circulation occurred from day 15 post challenge in mice vaccinated with CAF01-PyMSP1 whereas, in the alum adjuvanted PyMSP1 group, mice were not able to clear completely the parasites even at day 22 [262].

On the other hand, Tyagi and colleagues hypothesized that transdermal delivery may provide an alternative route for the administration of PfMSP1_19_ [232]. However, transdermal immunization results in poor immunogenicity due to the low permeability of hydrophilic antigens through the skin. To overcome this challenge, Tyagi and co-workers developed PfMSP1_19_ loaded in elastic or ultradeformable liposome vesicles composed of SPC and Span 80 (86: 14%, w/w). Thereafter, they evaluated the humoral and cell-mediated immune response in mice following the topical administration of the formulated elastic liposomes. Interestingly, these liposomes exhibited greater transcutaneous immunization, and induced robust and perdurable humoral response (specific IgG specific antibodies and isotypes, IgG1 and IgG3) in comparison with intramuscularly administered alum-adsorbed PfMSP1_19_ solution and topically applied PfMSP1_19_-loaded conventional liposomes. Moreover, when compared with other formulations, the elastic liposome-mediated topical delivery of PfMSP1_19_ achieved sizeable cell-mediated immune (CMI) response due to higher release of gamma interferon, a CMI activator factor that is a crucial player in conferring resistance to asexual blood stage malaria [232, 262, 263]. These results corroborated with those obtained previously after immunization with CAF01- PyMSP1 [262].

Additionally, immunization with mannosylated liposome vaccine formulation containing lipid core peptides as targeting ligands for the delivery of inactivated whole blood-stage parasite antigens (i.e. attenuated *P. chabaudi* or *P. yoelii*) to professional antigen presenting cells resulted in a significant induction of CD8 + T cell responses (adaptative immunity). Compared to control mice that received attenuated parasite extract in phosphate buffered saline (without liposomes), immunized mice demonstrated better control of parasitaemia as well as extended survival following challenges [264].

The 66PyIMP is an integral membrane protein isolated from the blood stages of *P. yoelii nigeriensis* multi-drug resistant strain [231]. Critical for the invasion of erythrocytes by the *Plasmodium* parasites (merozoites), this protein was exploited as target to elicit protective immune response in mice [265]. To screen for it potential to protect against malaria infection in mice, Sharma et al*.* [231] entrapped 66PyIMP in liposomal formulation containing EPC and cholesterol (2:1 molar ratio). The ability of these liposomes to generate cellular and humoral immune responses upon immunization in mice was compared with the emulsified form of 66PyIMP in incomplete Freund’s adjuvant (IFA) and free 66PyIMP. In vivo data indicated that, on day 6 post-infection, mice immunized with liposomal 66PyIMP exhibited 0.28% parasitaemia (i.e. 98% of chemosuppression) whereas IFA-66PyIMP-immunized mice and unimmunized mice showed 2.41% parasitaemia and 22.28% parasitaemia, respectively. Additionally, the group of mice immunized with liposomal 66PyIMP exhibited higher levels of IgG (IgG1 and IgG2a) in comparison with the rest of the experimental groups. The immunization of mice with conventional liposomes containing 66PyIMP was able to protect them against lethal *P. yoelii nigeriensis* infection (100% cured on day 20). In contrary, the survival time of mice immunized with free 66PyIMP was ranged between days 12 and 14 due to a late development of parasitaemia while the unimmunized mice died within 7–10 days [231].

#### Liposome-based vaccines for zygotes- and ookinetes-stage malaria

After administration of transmission-blocking vaccines (TBV), immunized patients may transfer induced antibodies to *Anopheles* mosquitoes during a blood meal thereby blocking the development of parasites in the mosquito gut [235]. Expressed on the surface of zygotes and ookinetes of *P. falciparum*, Pfs25 is a transmission-blocking vaccine candidate [266, 267]. However, clinical trials with Pfs25 contained in alum-based vaccines failed to produce satisfactory levels of antibodies [268, 269]. Hence, to address this weakness, Pfs25 was encapsulated in liposome-based adjuvants containing a core of biocompatible polymer that prevent its rapid release from the liposomal nanocarrier [270, 271]. Interestingly, following two intramuscular injections of the developed liposomal formulation in mice, strong Pfs25-specific antibody and Th1 cytokine responses were elicited for up to 8 weeks. Moreover, an even greater augmentation in these responses was observed after co-formulation of these gel core liposomes with CpG oligodeoxynucleotide (CpG-ODN), a Th1-type immune stimulant that is highly effective as adjuvant for vaccines [270, 271].

In another experiment, recombinant, polyhistidine-tagged (his-tagged) Pfs25 was mixed with liposomes containing DPPC, cholesterol, phosphorylated hexaacyl disaccharide (PHAD) and cobalt porphyrin-phospholipid (CoPoP) at a mass ratio of 4:2:1:1 [235]. PHAD is a synthetic monophosphoryl lipid A (MPLA) that acts as an immunostimulant, whereas CoPoP confers spontaneous nanoliposome antigen particleization (SNAP). Interestingly, SNAP immunization of mice and rabbits safely induced durable antibody responses with orders of magnitude greater than other adjuvants. Therefore, the SNAP approach constitutes a promising method for the development of recombinant vaccines that target different life-stages of the malaria parasites [235].

## Discussion: current scenario and perspectives

Due to its prevalence, severity and the complexity of its causative agent, malaria is one of the main public health concerns in poverty-ridden areas [1]. However, after one hundred years of using classical therapeutic approaches (drugs), the eradication of malaria remains a dream far from coming true, since malaria continues to be a clinical, social and economic burden. The issues concerning the current status of malaria partly include the physico-chemical (e.g. drug solubility and stability), pharmacokinetic (e.g. absorption and biodistribution), pharmacodynamics (e.g. potency and efficacy) and toxicological properties of the existing and emerging drugs [272, 273]. Interestingly, adequately designed drug liposomes can overcome some of these problems. Of note, liposomes are the most studied nanocarrier systems for malaria therapy and prevention because of its biodegradability, simple preparation method and versatility [274]. Moreover, liposomes have the capability of encapsulating one or more anti-malarial drugs on their surface (through electrostatic interactions), in their lipid bilayer (for lipophilic drugs) or in their aqueous core (for hydrophilic drugs) [10].

Since oral delivery of liposomes is hindered by their poor stability in the gastrointestinal tract and insufficient absorption across the intestinal epithelium [10, 99], all the anti-malarial liposome-based drug delivery systems developed until today were devoted for parenteral administrations. This constitutes a major limitation because oral drug delivery is the most preferred drug administration route due to many advantages. These include good patient compliance, convenience, cost-effectiveness, pain avoidance, ease of production, least sterility constraints, adaptability to accommodate various types of drugs and flexibility in the design of dosage form. Along with these undeniable benefits, there are also some defects of oral delivery route, like poor aqueous solubility, low penetration across intestinal membranes as well as potential chemical and enzymatic instability of drugs [275]. Hence, future research needs to be highly imaginative to develop liposomes (or liposome-type vesicles) for oral delivery of anti-malarial drugs through modulation of lipid composition, surface coating, thickening the interior aqueous phases, preparation of double liposomes and proliposomes, etc. [276–279]. Indeed, the incorporation of cholesterol and saturated lipids (e.g. DSPC) in the composition of liposomes are not sufficient enough to improve their in vivo stability and membrane rigidity in the gastro-intestinal tract. Nevertheless, parenteral administration of liposomes can be a useful weapon in case of severe malaria, or if the patients are vomiting and unable to take oral drugs [2, 9].

The encapsulation of anti-malarial drugs in conventional liposomes improved their in vivo efficacy against liver stage of malarial infections via passive targeting and allowed minimal dosing requirement, which limits the toxicity of the drugs in the tested animals. Nevertheless, to favour the lysosomotropism of these anti-malarial-encapsulating conventional liposomes, their size (< 100 nm) and charge (negative) constitute one of the main characteristics [108, 109, 115, 280]. While being affected by the size and number of bilayers, the amount of drug encapsulated in the liposomes also influences their efficacy [98]. On the other hand, it should be noted that, after opsonization with serum proteins in the blood circulation, the conventional liposomes administered by parenteral routes are rapidly cleared from the systemic circulation by the reticuloendothelial system (RES) [109, 274]. Hence, to overcome this phenomenon, conventional liposomes are usually coated with hydrophilic shields such as polyethylene glycols (PEG) [10, 106]. These inert and biocompatible hydrophilic polymers inferred steric stabilization to liposome surfaces as well as steric hindrance to the binding of ligand to the receptor thereby improving the pharmacokinetics of the liposomal formulations, prolonging their in vivo circulation time or controlling their drug release kinetics [274].

Additionally, drug-loaded PEGylated liposomes bearing a *P. berghei* amino acid sequence increased their targeting to liver without toxicity in the surrounding organs (heart, lungs and kidneys). However, due to the lack of sufficiently specific markers for *Plasmodium*-infected hepatocytes, liver-targeted liposomes have not progressed towards a strategy to fight malaria infection. As a consequence, most studies related to anti-malarial-loaded liposomes target the erythrocytic stage of malaria. Nevertheless, it should be noted that the very poor endocytic processes of RBCs and the lack of target selectivity against pRBCs and RBCs negatively affect the therapeutic potentials of both conventional and PEGylated liposomes as blood schizonticide [8, 281]. To counteract this weakness, liposomes are conjugated to pRBC- or RBC-directed surface ligands, antibodies, proteins or peptides. This approach represents the most-well studied liposomal strategy in malaria nanotherapy, and it led to improvement of anti-malarial efficacy in pre-clinical studies. Interestingly, pRBC- or RBC-targeted liposomes can deliver drugs before parasite infection and in the surrounding of RBCs infected with either *Plasmodium* early stages or *Plasmodium* late stages. The administration of a drug for each of these phases has its own advantages and challenges. Indeed, the delivery of drug-loaded liposome to RBCs before *Plasmodium* infection represents an interesting prophylactic approach. But it deserves further study since poor endocytic trafficking of liposomes in RBCs is one of the main obstacles limiting intracellular delivery. Other requirements have also to be considered such as (i) the concentration of immunoliposomes that avoid in vivo RBC agglutination, (ii) the potential interference of encapsulated drugs with the physiology of RBCs, and (iii) the possible removal of RBCs by the spleen due to aging or alterations induced by the adsorption of liposomes on their surface [8, 9].

On the other hand, the development of liposomes that specifically target the early blood stages of malaria has remained remarkably elusive. Indeed, the permeability of pRBCs to drugs is low until around six hours post-invasion. This implies that, within the six hours following invasion, only certain lipophilic drugs can enter RBCs, crossing different membranes located in parasitized RBCs, namely: (i) the plasma membrane of the erythrocyte, (ii) the parasitophorous vacuole membrane, (iii) the plasma membrane of the parasite and (iv) some additional lipid bilayers if the target is inside organelles (e.g. food vacuole or apicoplast) [177, 274]. The administration of drug-loaded liposomes to *Plasmodium* early erythrocytic stages would be an interesting option for eliminating the parasite before it completes its intraerythrocytic development. However, this strategy represents a challenging task [8].

In contrast, around 10–20 h post-invasion, significant alterations in the structural and functional characteristics of parasitized RBCs are observed thereby resulting in the formation of new permeation pathways and the entry of particles (nutrients, drugs) of less than 70 nm [8, 9, 175, 274]. Additionally, at this stage, the plasma membrane of parasitized RBCs become less elastic thus limiting the rebounding of randomly colliding liposomes and improving specific interaction of the targeting ligands with *P. falciparum* infected RBCs [8, 175, 281]. Indeed, a proof-of-concept study using quantum dots encapsulated in liposomes showed that the liposomal cargo entered the pRBCs through fusion of the liposome bilayer with the cell plasma membrane [8, 175, 281]. This fusion process is significantly dependent on the lipid composition and fluidity of the liposome membrane [274]. Thus, liposomes with relatively fluid lipid bilayers favour fusion events, but increase the risk of leaking for small drugs encapsulated in their aqueous core. In contrast, liposomes formulated with saturated lipids retain drugs better than with those formulated with unsaturated lipids thereby decreasing membrane merging with target pRBCs [98, 101, 179]. Moreover, it has been shown that immunoliposomes grafted with optimum range of PEG density and chain length elicit improved interaction with pRBCs as well as the liberation and internalization of sufficiently high amounts of their contents within *Plasmodium*-parasitized RBCs at the right time, in a safe and reproducible manner [8]. Based on this approach, liposome drug delivery systems applied to malaria therapy inhibit the parasite growth to a higher extent and minimize the development of resistant parasite strains [9]. Furthermore, parenteral liposomal vesicles may be adapted for individualized administration of new potent anti-malarial drugs having narrowed therapeutic windows, and specifically targeted them to pRBCs with good accuracy [282].

In addition to the above, it is noteworthy that, during their late erythrocytic stages (i.e. ca. 24 h after invasion), the *Plasmodium* parasites export a substantial number of receptors and transporters to the plasma membrane of host RBCs. Consequently, several other compounds will be able to cross the aforementioned membranes thanks to transporters or channels [8]. However, drug permeation through membranes of RBCs could be compromised by mutations in transporter molecules, a phenomenon that contributes to drug resistance in the malaria parasites [283, 284].

The in vitro and in vivo studies conducted so far have mainly focused on monotherapies with drugs facing resistance today (e.g. chloroquine), second-line anti-malarial drugs (e.g. primaquine) or drugs under development for the therapeutic management of malaria (e.g. monensin). Therefore, the development of liposomes based on anti-malarials currently available as well as the evaluation of their efficacy, safety, pharmacokinetics and tissue distribution constitute also a major avenue of future research. However, it should be noted that the WHO has recommended to malaria-endemic countries to phase out or halt the use of most of anti-malarial monotherapies [2]. Interestingly, the simultaneous administration of liposomes containing two or more drugs with similar or different pharmacokinetic profiles usually sustains constant relative ratios of drugs throughout the treatment schedule, thereby improving their single anti-malarial effects [129, 131]. Indeed, two drugs that typically display different plasma elimination rates may be subject to simultaneous plasma clearance when co-loaded in a single liposomal formulation [8, 9]. Of note, liposomes developed for combination therapy for malaria need to have reasonable long shelf half-lives. In fact, a general principle in ACT is that the partner drug (or second drug) should have a longer half-life than the artemisinin derivatives (i.e. primary drug) to eliminate the residual parasite not cleared by the latter. However, concentrations of the partner drug that remain below threshold levels in the days following treatment with artemisinin-based combinations need to be monitored carefully when tackling the issue of drug resistance development. Therefore, anti-malarial drugs with short half-lives (e.g. artemisinin derivatives and curcumin) make good candidates for co-delivery using liposomes for combination therapies [82, 129, 131, 285–287].

Despite these various scientific advances made to date, many efforts are ongoing to identify new targeting agents, which can promote anti-malarial activity, enabling selective and controlled release as well as enhanced cell penetration (to deliver a lethal amount of anti-malarial agent to the right place). Indeed, future targeted delivery strategies would be susceptible of adaptation by taking into account the elevated antigenic variability of the parasite, the other human (e.g. gametocytes) and mosquito stages (e.g. gametes, ookinetes and oocysts) of parasites as well as the different *Plasmodium* species (e.g. *P. ovale, P. vivax* and *P. malariae*) [288].

The in vitro antiplasmodial activity of anti-malarial-loaded liposomes was mainly evaluated on blood stages of *P. falciparum* laboratory strains (chloroquine-sensitive, chloroquine-resistant and chloroquine-multidrug resistant) using the titrated [^3^H]-hypoxanthine incorporation method. However, it would also be interesting to screen the antiplasmodial sensitivity of these liposomes against the erythrocytic stages of others *Plasmodium* species as well as clinical isolates. Moreover, the ability of drug-encapsulated liposomes to cure the liver stage infections could be evaluated by the type II fatty acid synthase (FAS)-target-based anti-malarial screening approach or the real time measurements of bioluminescence of in vitro cultured liver stages [83, 97, 289].

To assess the therapeutic in vivo response of anti-malarial compounds loaded in liposomes, rodent models were typically used. These models have been validated and extensively used in different aspects of research on the human infection and are recognized as useful model parasite for evaluation of in vivo anti-malarial efficacy of drugs, in particular for both liver and blood stages of uncomplicated falciparum malaria [290]. Indeed, the rodent *Plasmodium* (*P. berghei*, *P. chabaudi*, *P. vinckei* and *P. yoelii*) have shown some analogies with human parasites such as basic biology (genome organization, metabolic pathway), life cycle and the molecular basis of drug-sensitivity and resistance. Nevertheless, the efficacy of liposomal formulations against *P. vivax* malaria should also be investigated by using *Plasmodium cynomolgi* and *Aotus* monkeys, an appropriate host-parasite combination [291, 292]. Of note, the *Plasmodium* mouse model of experimental cerebral malaria does not replicate exactly the pathophysiology of cerebral disease in humans [291–293]. Even though only few in vivo studies were dedicated to cerebral malaria, caution is required in the extrapolation of data from experimental cerebral malaria to human cerebral malaria. Indeed, in contrast to human cerebral malaria, experimental cerebral malaria is not associated with important sequestration of cytoadherent mature trophozoite or schizont infected red blood cells in brain vessels. Moreover, human cerebral malaria shows both clinical heterogenicity between patients and the patterns of pathophysiology observed between adults and children [292]. Hence, the efficacy of liposome-based drug delivery systems in cerebral malaria should be investigated in different animal models including non-human primate models that are more relevant [294]. Identification of new biosensors/biomarkers and their couplage with targeting ligands will be a promising and attractive strategy for the development of theranostic liposomes.

From 1980 to 2020, the term liposomes had scored in more than 66,000 publications (Pubmed database). However, over the same period, less than 200 articles (i.e. around 5 articles per year) devoted to liposomes applied to malaria therapeutics have been identified. These articles were produced by less than 20 research groups, which appear to be far lower than those dedicated to “liposome for cancer” (ca. 6000 articles in 2020 only plus 300 cancer research groups in the world; Pubmed database); thereby confirming that liposome technology for malaria is still an under-researched topic. Additionally, one can argue that liposome-based administration has not progressed yet towards a working strategy for malaria therapeutics. Indeed, among the 19 clinically approved liposomal formulations, none is indicated for malaria therapy, probably because of the highly variability of known *Plasmodium* proteins found in the plasma membrane of pRBCs and the lack of sufficiently specific markers for *Plasmodium*-infected cells [9]. On this note, liposome nanotechnology looking into the development of *Plasmodium* biomarkers makes a great avenue for future research. On the other hand, numerous liposomal vaccines for malaria prophylaxis have shown promising results in pre-clinical studies, however, only a handful of candidate vaccines have made it to clinical trials. Among them, RTS,S/AS01 (Mosquirix^®^) has been approved in 2015 by the European Medicines Agency for immunization of children aged 6 weeks to 17 months against *Plasmodium* malaria. However, this vaccine provides relatively little protection and its efficacy decreases rapidly [30, 255, 295, 296]. Hence, significant efforts are to be made to develop ideal malaria vaccines that have the ability of inducing both humoral and cell-mediated immune responses as well as sterile immunity against the different life-cycle stages of malaria [30]. The development of these multi-stage vaccines may require appropriate and/or innovative liposome-based adjuvants [297].

Future efforts are to be dedicated to the evaluation of the safety profile of liposomes and the toxicity of their material components (e.g. ligands) prior to clinical translation [298]. The distribution and localization of liposomes after their administration are also critical aspects that have to be ascertained to realize their clinical potentials. The stability of liposome-based medicines upon storage or in vivo has to be evaluated to fill the knowledge gap in this field. From the perspective of the pharmaceutical industry, numerous formulation parameters, such as drug loading capacity, stability, sterilization, batch reproducibility and scale up protocols have to be carefully considered [104, 106, 299]. The application of good manufacturing practice for the functionalization of liposome surface and others manufacturing techniques are also crucial. Beyond these pharmaceutical manufacturing limitations, cost-related issues (industrial interest, cost production, public and private economic efforts), government regulations and intellectual property are to be taken into account as well to boost the clinical translation of liposome-based anti-malarial treatments.

Finally, despite their very limited number and the numerous pharmacological, pharmacotechnical and economic challenges, the studies related to anti-malarial liposomes evidenced some potential for the treatment of malaria considering the significant therapeutic advantages of liposomal formulations over conventional drug products.

## General conclusion

The potentials of liposome technology as a technique for building personalized tools to treat and prevent malaria by overcoming the limitations associated with existing and developing anti-malarial medicines and antigens are demonstrated in this overview of works published between 1980 and 2020. The shortcomings of these therapeutic and immunological agents include poor solubility, low permeability, chemical instability, low bioavailability, uncontrolled pharmacokinetics, undesirable side effects, toxicity, development of resistance, non-specific targeting and poor immunogenicity. The impacts of passive targeting liposome systems and antibody-mediated erythrocytes targeting liposomes as well as liposome-based vaccines have been demonstrated both in vitro and in vivo using various pre-clinical settings. However, these promising performances are still in their infancy due to the lack of extensive investigations, as most studies are focused on testing liposomes against one *Plasmodium* species from a single source (mostly laboratory strains), instead of considering a systematic assessment using various species from multiple origins (e.g. clinical isolates). In addition, complementary studies (e.g. physico-chemical stability, scaling up, sterilization process, toxicology, oral delivery) as well as key prerequisites (e.g. production cost, social impact) are needed to fully realize the potential of liposome technology for malaria as a tropical disease, which will further boost the translational development of anti-malarial liposomes towards clinical settings.

## Data Availability

Not applicable.

## Abbreviations

ACT: Artemisinin-based combination therapy
ALF: Army liposome formulation
ALFA: ALF plus alum
ALFQ: ALF plus QS-21
anti-mRBC: Anti-mouse RBCs
CHEMS: Cholesteryl hemisuccinate
C_max_: Maximum plasma concentration
CoPoP: Cobalt porphyrin-phospholipid
CSP: Circumsporozoite protein
DBPC: Dibehenoylphosphatidylcholine
DEPC: Dierucoyl phosphatidylcholine
DMPC: Dimyristoylphosphatidylcholine
DMPG: Dimyristoylphosphatidylglycerol
DOPC: Dioleoylphosphatidylcholine
DOTAP: 1,2-Dioleoyl-3-trimethylammoniumpropane
DPPC: Dipalmitoylphosphatidylcholine
DPPG: Dipalmitoylphosphatidylglycerol
DSPC: Distearoylphosphatidylcholine
DSPE: Distearoylglycerophosphoethanolamine
DSPE-mPEG2000: Distearoylglycerophosphoethanolamine-methoxy-polyethylene glycol 2000
EPC: Egg phosphatidylcholine
EPG: Egg phosphatidylglycrol
FDA: Food and Drug Administration
GPA: Glycophorin A
HSPC: Hydrogenated soybean phosphatidylcholine
ICG: Indocyanine green
LPS: Lipopolysaccharides
Mal: Maleimide group
MPB-PE: Maleimido-4-(*p*-phenylbutyrate)phosphatidylethanolamine)
MPB-PE-REV: Maleimido-4-(*p*-phenylbutyrate)phosphatidylethanolamine reverse-phase evaporation vesicles
MPLA: Monophosphoryl lipid A
MPS: Mononuclear phagocytic system
nSSL: Nanosterically stabilized liposomes
PC: Phosphatidylcholine
PE: Phosphatidylethanolamine
PEG: Polyethylene glycol
PfEMP1: *P. falciparum* Erythrocyte membrane
PG: Phosphatidylglycrol
PHAD: Phosphorylated hexaacyl disaccharide
PL: Phospholipon
POPC: Palmitoyloleoylphosphatidylcholine
pRBC: Parasitized red blood cell
PS: Phosphatidylserine
QS-21: *Quillaja saponaria* Molina, fraction 2
RBC: Red blood cell
SPC: Soybean phosphatidylcholine
T_max_: Time to maximum plasma concentration
WHO: World Health Organization
